# Supplementary material for: Integrating fragment-based screening with targeted protein degradation and genetic rescue to explore eIF4E function
Source: Nat Commun. 2024 Nov 20;15:10037. doi: 10.1038/s41467-024-54356-1 (PMC11868579; doi:10.1038/s41467-024-54356-1)
Supplement: Supplementary file 1 — Supplementary Information [file 41467_2024_54356_MOESM1_ESM.pdf]

Integrating fragment-based screening with targeted protein degradation and genetic rescue to explore eIF4E function

Swee Y. Sharp, Marianna Martella, Sabrina D'Agostino, Christopher I. Milton, George Ward, Andrew J. Woodhead, Caroline J. Richardson, Maria G. Carr, Elisabetta Chiarparin, Benjamin D. Cons, Joseph Coyle, Charlotte E. East, Steven D. Hiscock, Carlos Martinez-Fleites, Paul N. Mortenson, Nick Palmer, Puja Pathuri, Marissa V. Powers, Susanne M. Saalau, Jeffrey D St. Denis, Kate Swabey, Mladen Vinković, Hugh Walton, Glyn Williams, Paul A. Clarke

1. **SUPPLEMENTARY FIGURES AND LEGENDS**
2. **SUPPLEMENTARY TABLES AND LEGENDS**
3. **SUPPLEMENTARY NOTES: COMPOUND SYNTHETIC METHODS, INCLUDING ANALYTICAL CHARACTERISATION AND EXPERIMENTAL PROCEDURES FOR THE MEASUREMENT OF CHROMLOGD, AQUEOUS SOLUBILITY, PLASMA PROTEIN BINDING, CACO2 PERMEABILITY AND MICROSOMAL CLEARANCE.**
4. **SUPPLEMENTARY REFERENCES**

1. SUPPLEMENTARY FIGURES AND LEGENDS

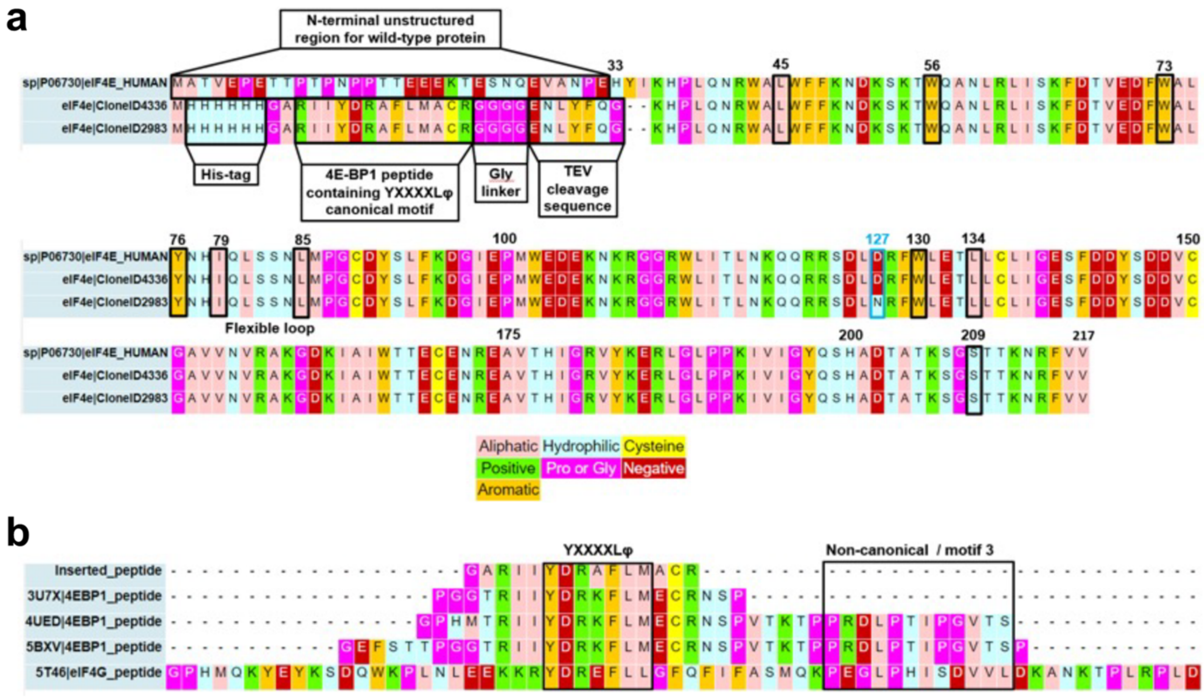

Supplementary Figure 1. eIF4E, 4E-BP1 and eIF4G protein peptide sequences. a eIF4E protein sequence alignments. P06730 is the consensus sequence from UniProt. CloneID2983 is the sequence of the engineered protein based on the minor variant (N127) which was used for X-ray crystallographic fragment screening. CloneID4336 is identical to 2983 except for D127 (coloured blue); The figure is annotated with key features: Residues 1-32 are unstructured and not visible in the PDB structure 3U7X; Residues numbered and highlighted in boxes were selected for mutational analysis (see Supplementary Table 2) , other residues are numbered to aid the reader; His-tag (6 histidines, for purification purposes); The short 4E-BP1 peptide contains the canonical PPI binding sequence YXXXXLφ and corresponds to YDRAFLM in the figure; TEV cleavage sequence = Tobacco etch virus protease cleavage sequence. Gly linker – flexible glycine linker connecting canonical peptide to eIF4E. Residues are colour coded by properties according to the key. **b** 4E-BP1 and eIF4G peptide sequences (from PDB structures 3U7X, 4UED, 5BXV, 5T46) aligned with the sequence of the peptide inserted into the engineered protein.

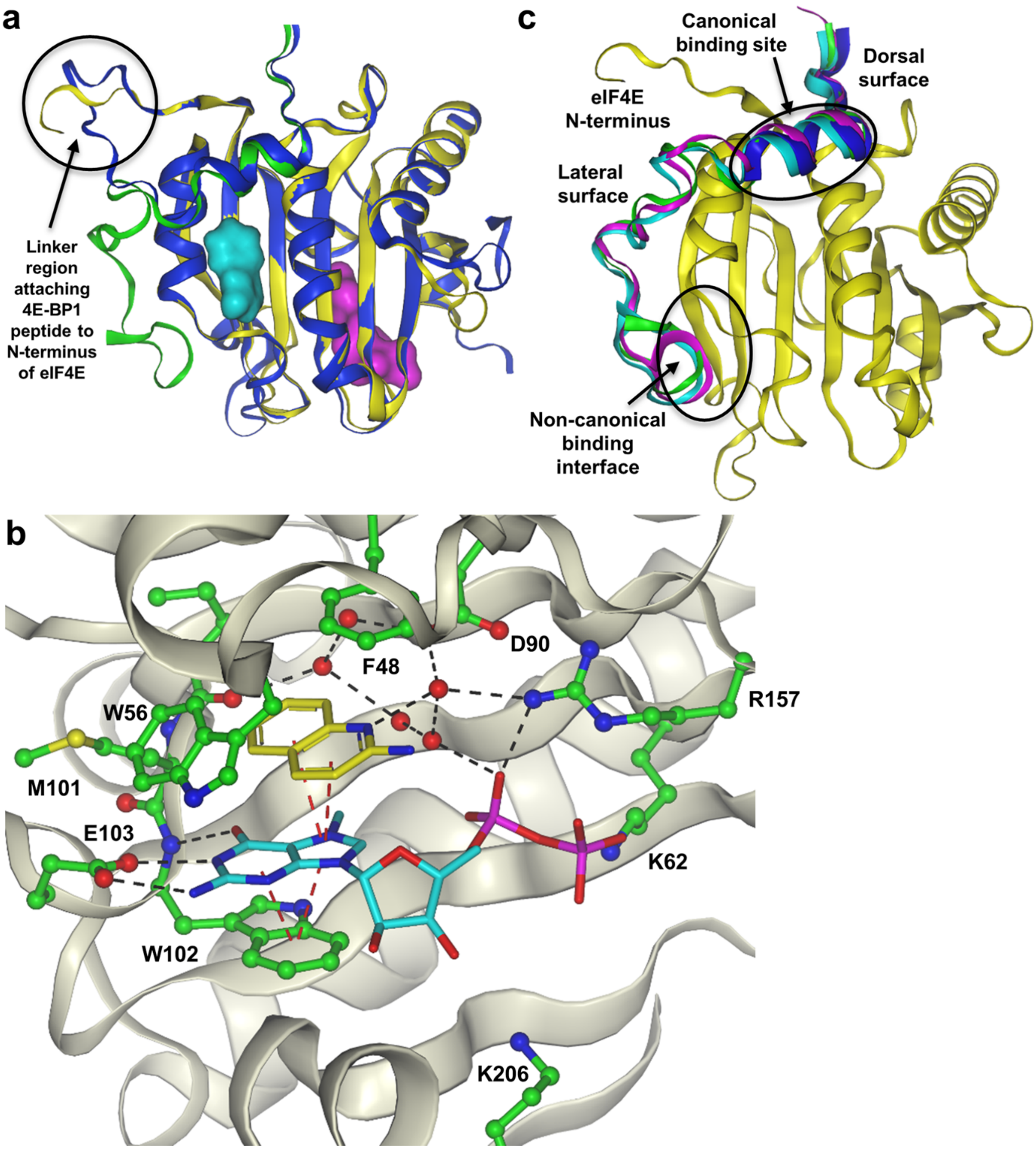

Supplementary Figure 2. Comparison of wild type and engineered secondary structure, comparison of 4E-BP1 / 4G proteins and a magnified view of fragment hit in the Cap site. a Overlay of the secondary structures of wild type eIF4E (yellow), the engineered protein used for fragment screening (blue ribbon) and a fragment of eIF4G (green ribbon). The mRNA cap-binding site is highlighted with a Connolly surface representation of m7-GTP (magenta) and site 2 is highlighted with the Connolly surface of compound **1** (cyan). **b** 2-Aminoquinoline (yellow) discovered through fragment screening bound cooperatively with m7-GDP (cyan) at the Cap site of eIF4E. Residues within 4Å of the two ligands are shown in ball and stick

representation. Water molecules are displayed as red spheres, hydrogen bonding and π-π interactions are displayed as black and red hashed lines respectively. **c** Overlay of the secondary structure of wild type eIF4E from PDB structure 5T46 (yellow), with the secondary structures of various binding partners: eIF4G peptide from 5T46 (green) and 4E-BP1 peptides from 3U7X (blue), 4UED (magenta) and 5BXV (cyan) respectively.

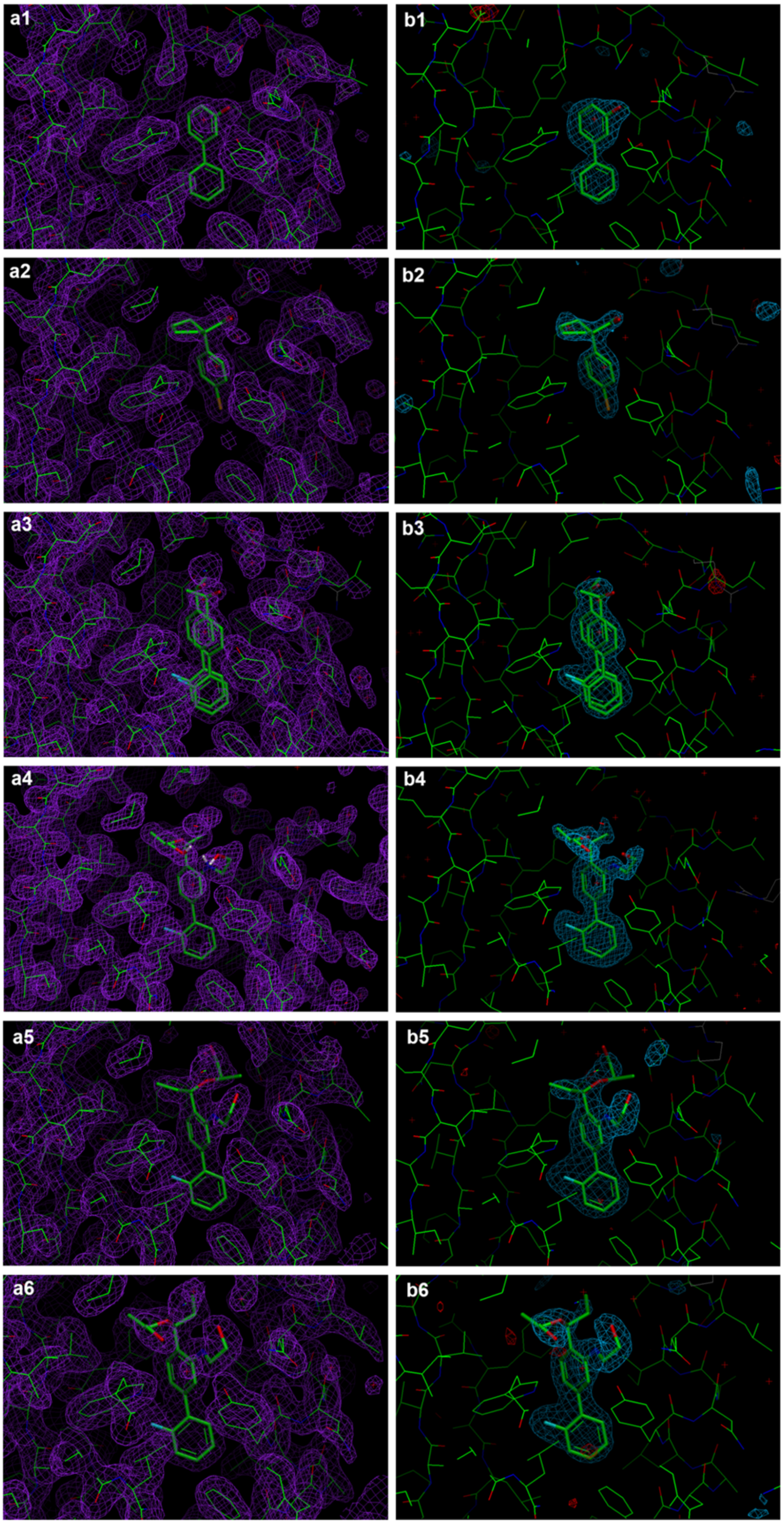

Supplementary Figure 3. Electron density maps for compounds 1-5. a1-5 2Fo-Fc

maps for the minor variant of eIF4E are shown as a purple mesh contoured at sigma level 1. **b1-5** Omit maps for the minor variant of eIF4E are shown as a blue mesh and are contoured at sigma level 3.5 for compounds **1**, **2**, **5** and sigma level 4 for compounds **3** and **4**. **a6** and **b6** are the 2Fo-Fc (purple mesh, sigma level 1) and omit maps (blue mesh, sigma level 3.5) respectively of compound **5** bound to the major variant of eIF4E.

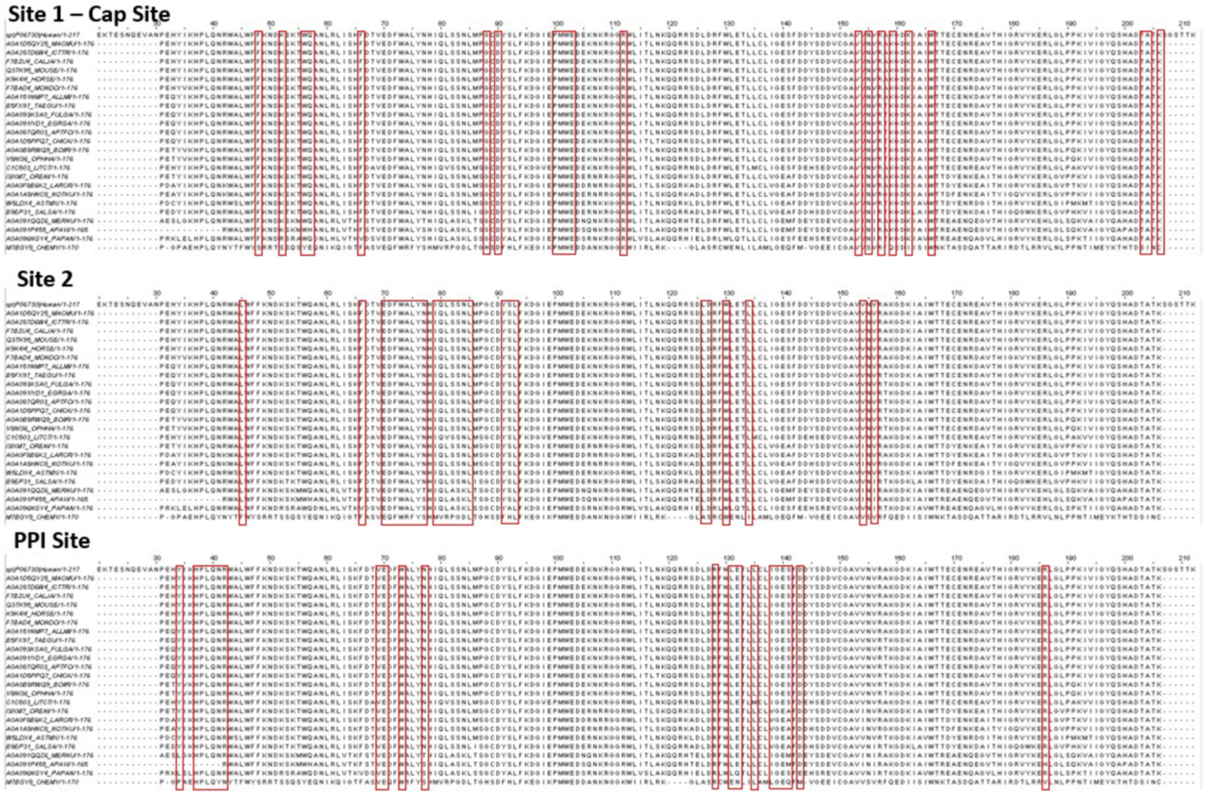

Supplementary Figure 4. Sequence alignment of human eIF4E (from Uniprot code P06730) and representative ortholog sequences of eIF4E. The 20 residues defining each of the sites which were used for the OrthoCons sequence analysis (Supplementary Fig. 5) are highlighted in red.

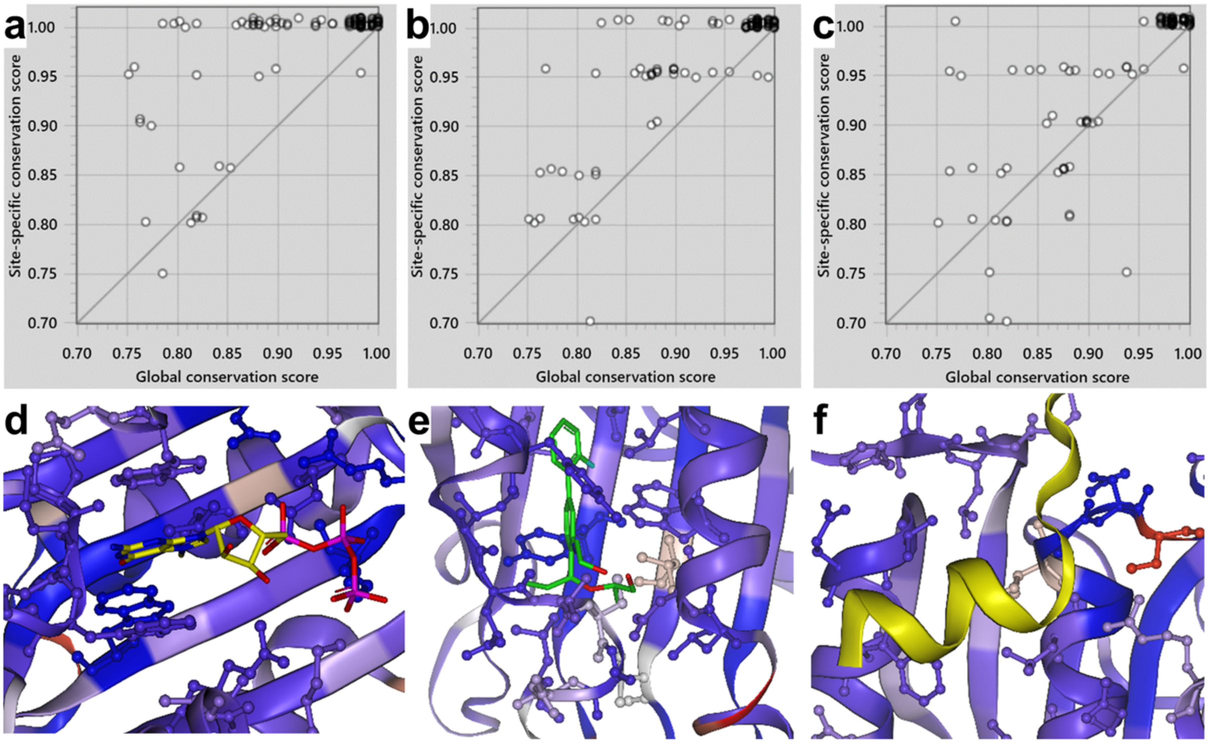

Supplementary Figure 5. Analysis of cross species sequence conservation. Site specific vs global sequence conservation for 115 orthologues of eIF4E analysed by OrthoCons (Astex developed software) and plotted using the PDB structure 5T46 of human eIF4E for **a** site 1 using m7-GTP (Cap-site), **b** site 2 using compound **4**, **c** canonical binding site using 4E-BP1 derived peptide (from PDB structure 3U7X). Points above the diagonal indicate the site is more highly conserved than the global sequence. **d, e, f** shows respectively the 3 different binding sites with the ligand or peptide displayed in yellow or green. Sequence conservation is highlighted by colour. Blue is high, white is medium and red is low conservation.

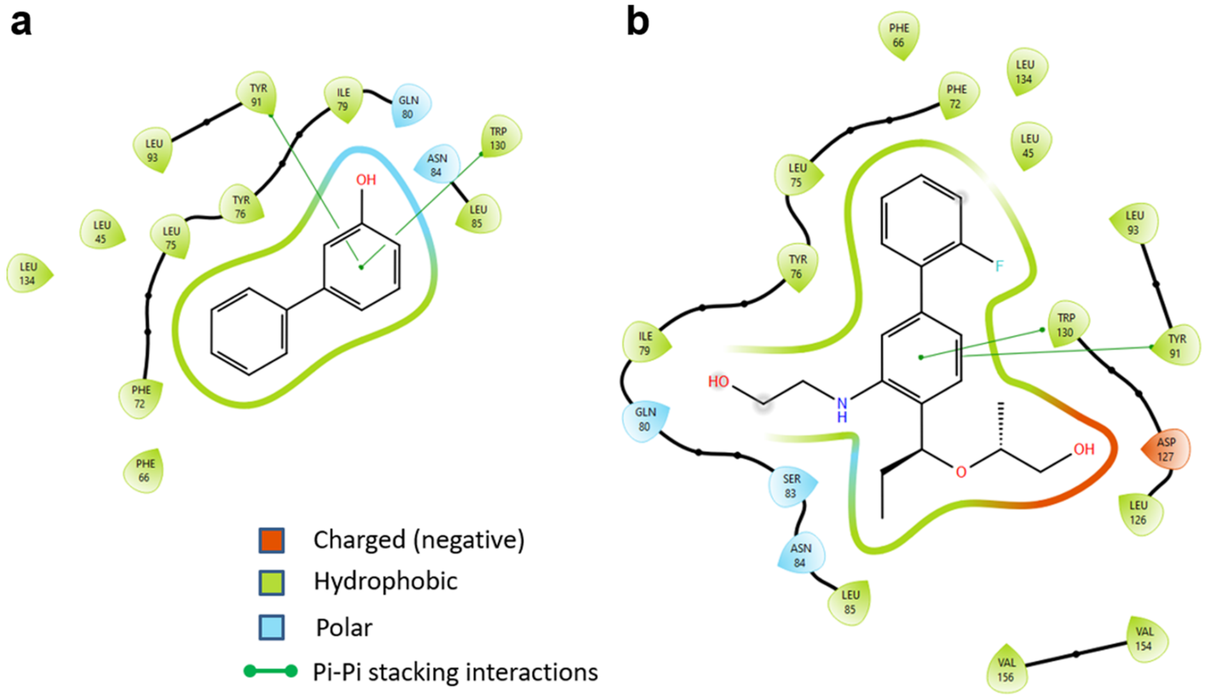

Supplementary Figure 6. Protein-ligand interaction diagrams. **a** Depicting the major interactions between eIF4E and Compound 1 and **b** Compound 4. 2D-ligand interaction diagrams were generated in Maestro, Schrodinger 2023 (Schrödinger Release 2023-1: Maestro; Schrödinger, LLC, New York, NY, USA).

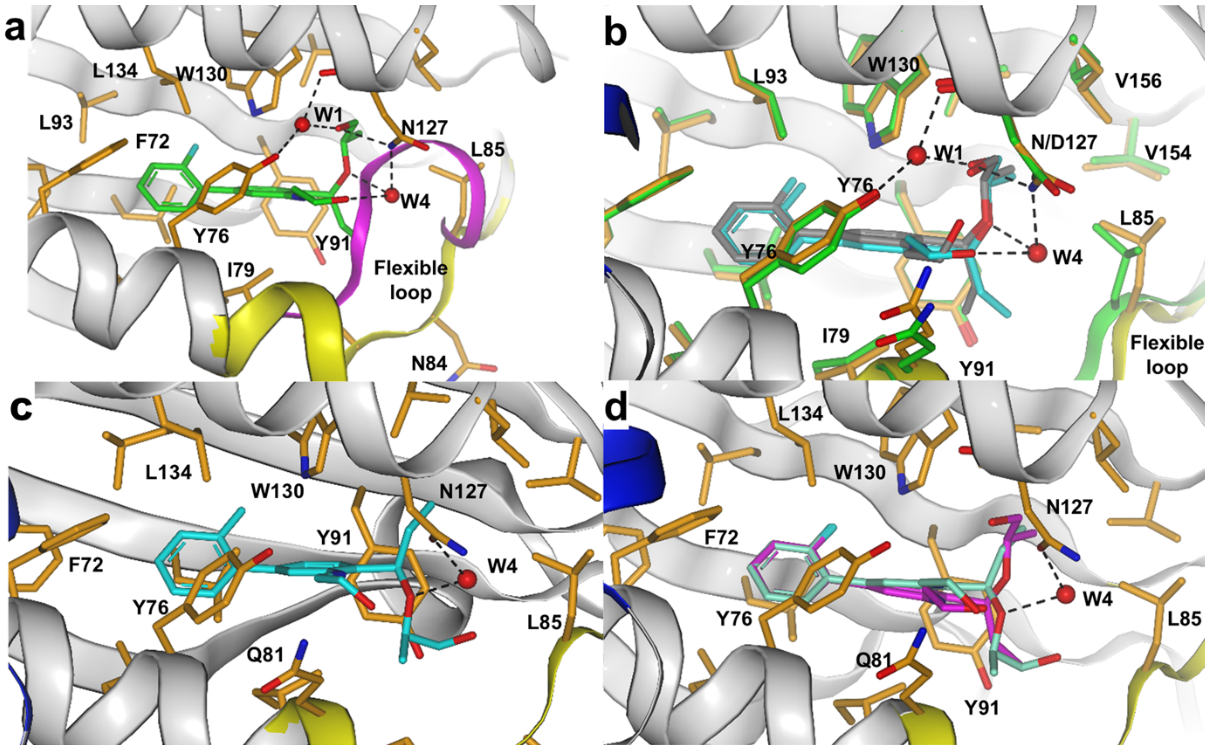

Supplementary Figure 7. Protein-ligand co-crystal structure comparisons of tool compound 4, its less active diastereoisomer, compound 5. a Crystal structure of compound **4** overlaid with the flexible loop conformation from initial fragment hit (compound **1**). **b** Compound **4** bound to the D127 and N127 variants. Compound **4** is shown in cyan for N127 and grey for D127. Protein residues are coloured orange for N127 and green for D127, the flexible loop region is yellow (N127) and green (D127). **c** Compound **5** bound to eIF4E (N127). Compound **5** is displayed in cyan, residues within 4Å of the ligand are orange has rotated by approximately 180° to accommodate ligand binding, resulting in the loss of key polar interactions. The highly conserved water (W1) is no longer present in the structure. **d** Overlay of compounds **4** and **5**. **4** is coloured pink, **5** is coloured cyan, the protein structure is coloured as previously described.

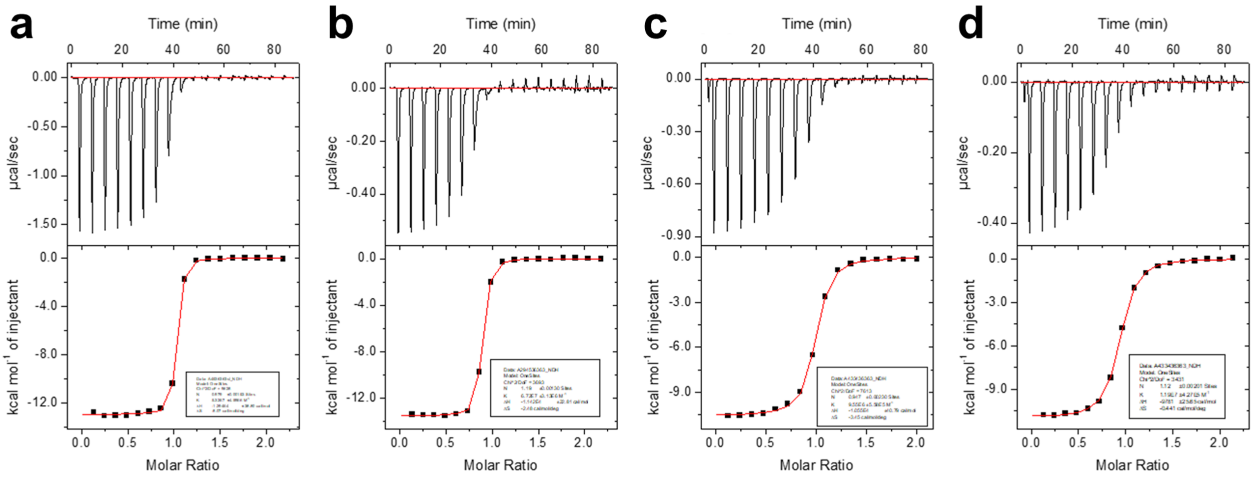

Supplementary Figure 8. Standard and reverse ITC titrations of compound 4 binding to eIF4E D127N and eIF4E D127. a Standard ITC titration of eIF4E D127N with compound 4. **b** Reverse ITC titration of compound 4 with eIF4E D127N. **c** Standard ITC titration of eIF4E D127 with compound 4. **d** Reverse ITC titration of compound 4 with eIF4E D127. Binding of compound 4 to eIF4E D127N was driven by a large favourable enthalpic contribution (ΔH = -12.0 ± 0.5 Kcal/mol) with a small entropic penalty ($-T\Delta S$ = 1.4 ± 0.5 Kcal/mol). Binding of compound 4 to eIF4E D127 showed a similar thermodynamic profile, with a large favourable enthalpy (ΔH = -10.2 ± 0.4 Kcal/mol) and a small entropic penalty ($-T\Delta S$ = 0.6 ± 0.4 Kcal/mol).

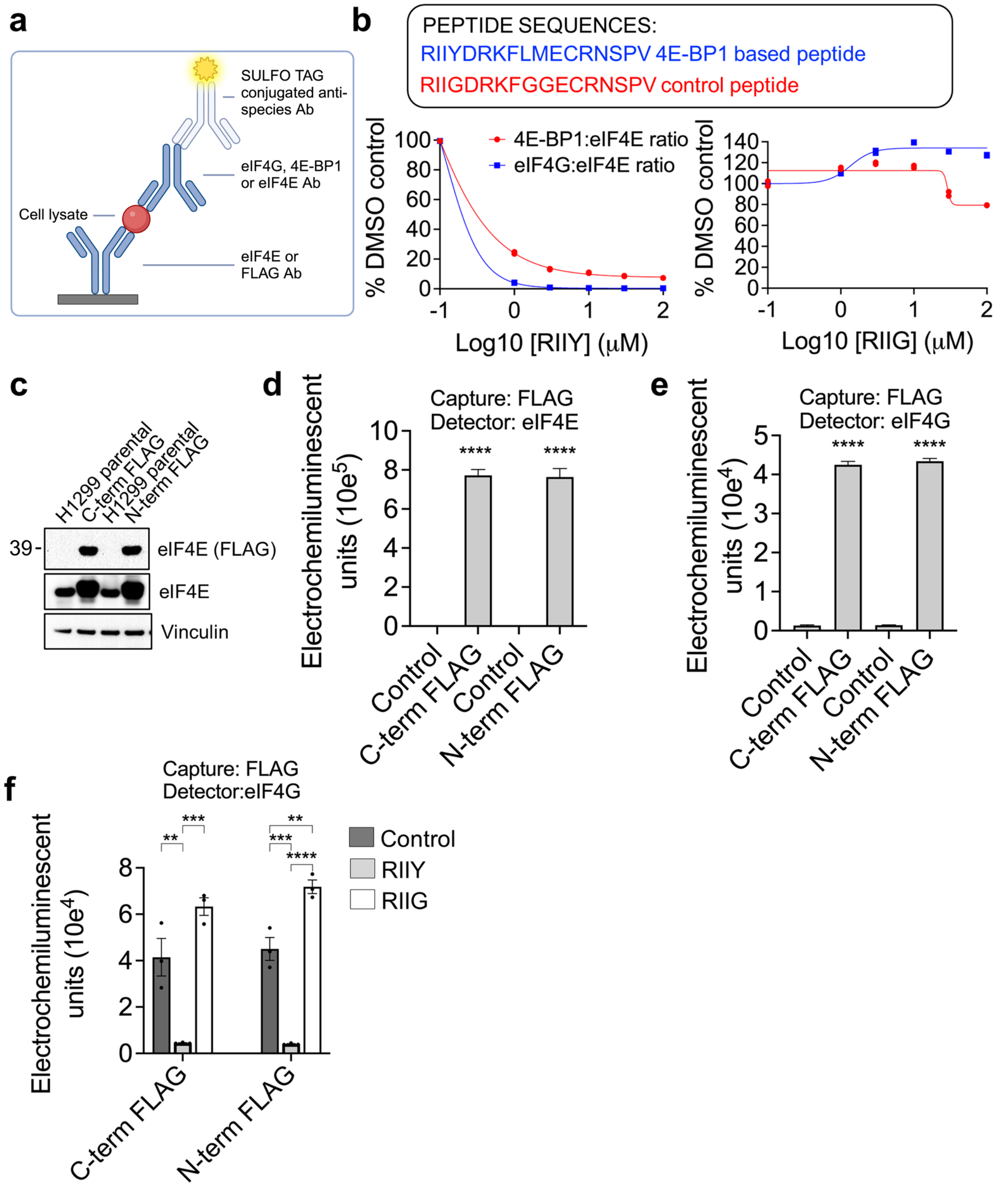

Supplementary Figure 9. Electro-chemiluminescent eIF4E: 4E-BP1 and eIF4E:eIF4G binding assay in H1299 human non-small cell lung cancer cells. a Scheme of the electro-chemiluminescent assay for quantification of eIF4E expression or eIF4E:eIF4G interaction (created in BioRender. Powers, M. (2024) https://BioRender.com/v30a057). Plates coated with anti-eIF4E or anti-FLAG antibody were used to capture endogenous eIF4E or exogenous FLAG-tagged eIF4E from cell lysates. eIF4E-bound EIF4G or 4E-BP1 were detected with their respective antibodies

and normalised for expression with an eIF4E antibody. **b** Sequences of the RIIY 4E-BP1 derived positive control peptide or RIIG negative control peptide. Electro-chemiluminescent assay for binding of 4E-BP1 or eIF4G with endogenous eIF4E in SW620 lysates following incubation for 30 min with DMSO control, and 1-100 µM RIIY (left plot) or RIIG (right plot) peptides. Results show individual luminescence signals from 2 biological replicates. **c** Immunoblot of eIF4E and FLAG-tagged eIF4E in H1299 cells expressing C- or N-terminal FLAG-tagged eIF4E (n = 3 biological replicates). Vinculin was used as a loading control. **d** Quantification of exogenously expressed C- or N-terminal FLAG-tagged eIF4E or **e** eIF4E binding to eIF4G in H1299 cells by electro-chemiluminescent assay. Untransfected H1299 cells were used as controls. Each data point represents the mean luminescence signals ± SD from 3 biological replicates. p values were <0.0001 for both control vs C- or N-terminal FLAG-tagged eIF4E or eIF4E binding to eIF4G. **f** Electro-chemiluminescent quantification of the binding of C- or N-terminal FLAG-tagged eIF4E to the eIF4G after addition of the competitive RIIY 4E-BPI derived peptide or RIIG negative control peptide (100 µM) or vehicle only (control) to H1299 cell lysates for 30 mins. Each data point represents mean luminescence signals ± SEM from 3 biological replicates. Significant p values (<0.05) for C-term binding to RIIY were 0.0056 (control vs RIIY), and 0.0005 (RIIY vs RIIG). Values for N-term binding to RIIY were 0.0003 (control vs RIIY), 0.003 (control vs RIIG) and <0.0001 (RIIY vs RIIG). For all plots an ordinary one-way ANOVA with Tukey multiple comparisons test was performed with comparison to controls, and source data is located in the Supplementary Source Data file. For all plots p values are denoted as $^{*}p<0.05$, $^{**}p<0.01$, $^{***}p<0.001$, $^{****}p<0.0001$.

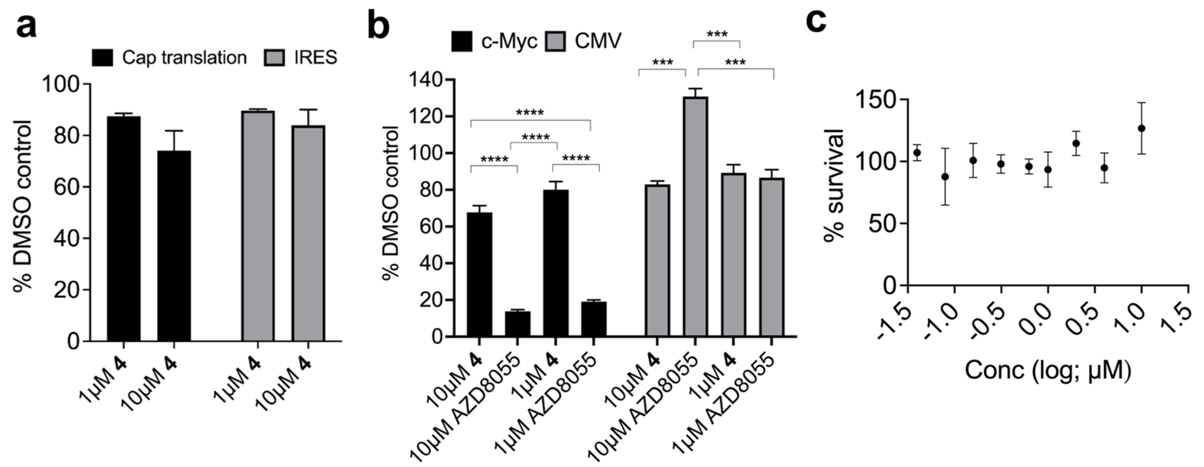

Supplementary Fig. 10 Determination of the effects of compound 4 on protein synthesis and viability. a HEK293 cells were transfected with protein synthesis reporter expressing bicistonic mRNA with a cap-dependent luciferase reporter and a cap-independent luciferase driven by a viral IRES. Cells were treated with 1 or 10 μM with compound **4** for 24 hr. Luminescence was measured using the Dual-glo luciferase assay (mean ± SEM from 3 biological replicates). **b** SW620 cells were transfected with a c-Myc-regulated firefly luciferase expression reporter or a constitutive CMV promoter-regulated renilla luciferase reporter. Cells were treated for 24 hr with 1 or 10 μM compound **4** or the mTORC2 kinase inhibitor AZD8055 as positive control (mean ± SEM from 3 biological replicates). An ordinary one-way ANOVA with Tukey multiple comparisons test was performed comparing c-Myc-regulated firefly luciferase expression or CMV-regulated Renilla luciferase expression following the different treatments. For c-Myc-regulated firefly luciferase expression, all significant p values were <0.0001. For CMV-regulated Renilla luciferase expression, p values were 0.0001 (10 μM compound 4 vs 10 μM AZD8055), 0.0003 (1 μM compound 4 vs 10 μM AZD8055) and 0.0002 (10 μM AZD8055 vs 1 μM AZD8055). **c** Cell viability measured by cell titre blue assay was performed in SW620 cells treated with compound **4** for 4 days (mean ± SEM from 3 biological replicates). Source data is located in the Supplementary Source Data file

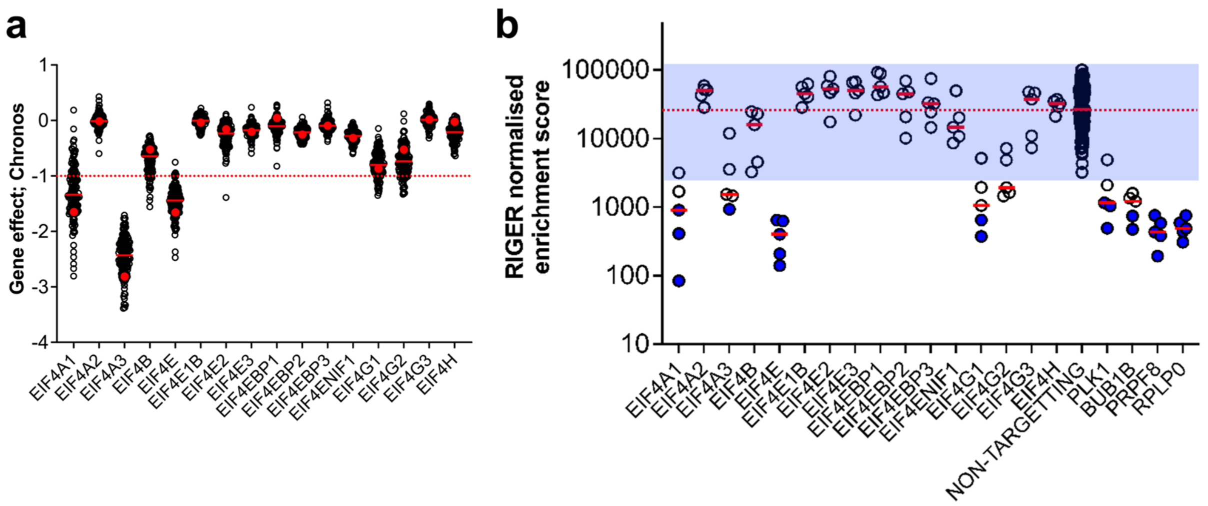

Supplementary Fig. 11 Dependency of human H1299 NSCLC cells on the components of the eIF4F protein synthesis initiation complex. a Summary plot of DEPMAP (https://depmap.org/portal/) shRNA gene scores for eIF4F complex components or interactors (H1299 cells - red open symbols) in lung cancer cells. **b** H1299 NSCLC cells were transduced with a lentiviral whole genome shRNA library with 8 shRNAs per gene target. Samples were taken 3 days after viral transduction and selection (day 0) and after a further 21 days in culture. Genomic DNA was extracted from the transduced cells and surviving shRNA constructs identified using next generation sequencing. The significant ($p<0.05$) depletion or enrichment of the shRNAs was determined using RNAi gene enrichment ranking (RIGER). The shaded area encompasses the enrichment scores (NES) range for the non-targeting control shRNAs, the red-dotted line indicates the median score for the non-targeting controls. Each point represents the NES for a single repeat (data for 5 biological replicates are plotted). Increased dependency is associated with a decreased NES score. Four essential genes (*PLK1*, *BUB1B*, *PRPF8* and *RPLP0*) that cells are dependent on were included as positive controls (blue filled symbols - $p< 0.001$), source data is located in the Supplementary Source Data file.

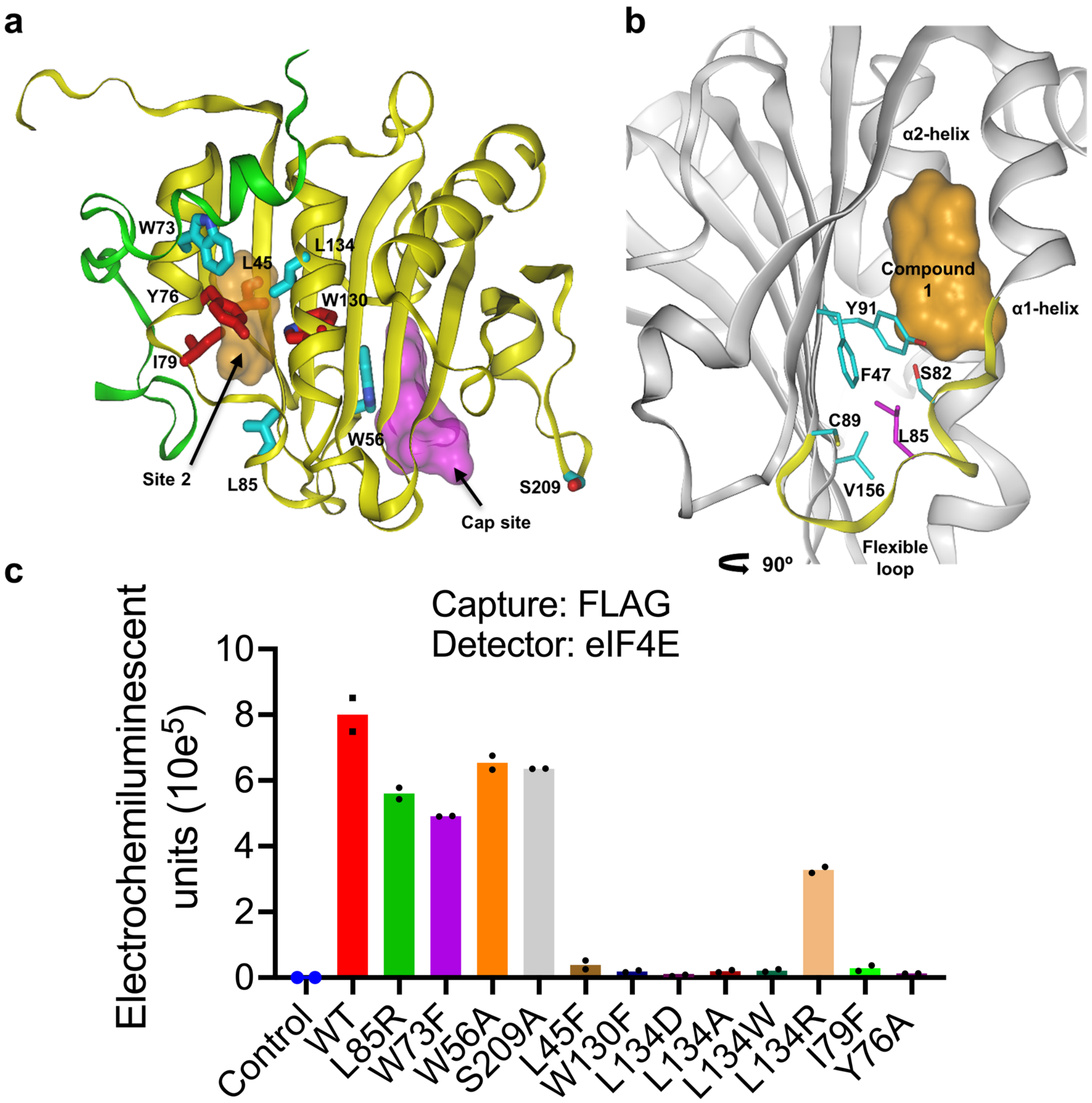

Supplementary Fig. 12 Location of residues selected for mutation and expression of the N-terminal FLAG-tagged eIF4E transfected with wild-type eIF4E or a series of eIF4E mutants. a X-ray crystal structure of eIF4E protein overlaid with eIF4G peptide as shown in Fig. 1a. The location of residues selected for mutational analysis are highlighted in cyan (acceptable expression) and red (low expression). **b** Loop region of eIF4E. L85 is highlighted in purple and sidechains of amino acid residues within 4Å of Leu 85 are shown in cyan. The Connolly surface of compound **1** (orange) is included to highlight site 2. **c** Quantification of N-terminal FLAG-tagged eIF4E expression determined by the electro-chemiluminescent assay in H1299 control untransfected or H1299 cells transfected with, wild-type (WT) eIF4E or

a series of eIF4E mutants. Data shown are the mean luminescence signals from n = 2 biological replicates, source data is located in the Supplementary Source Data file.

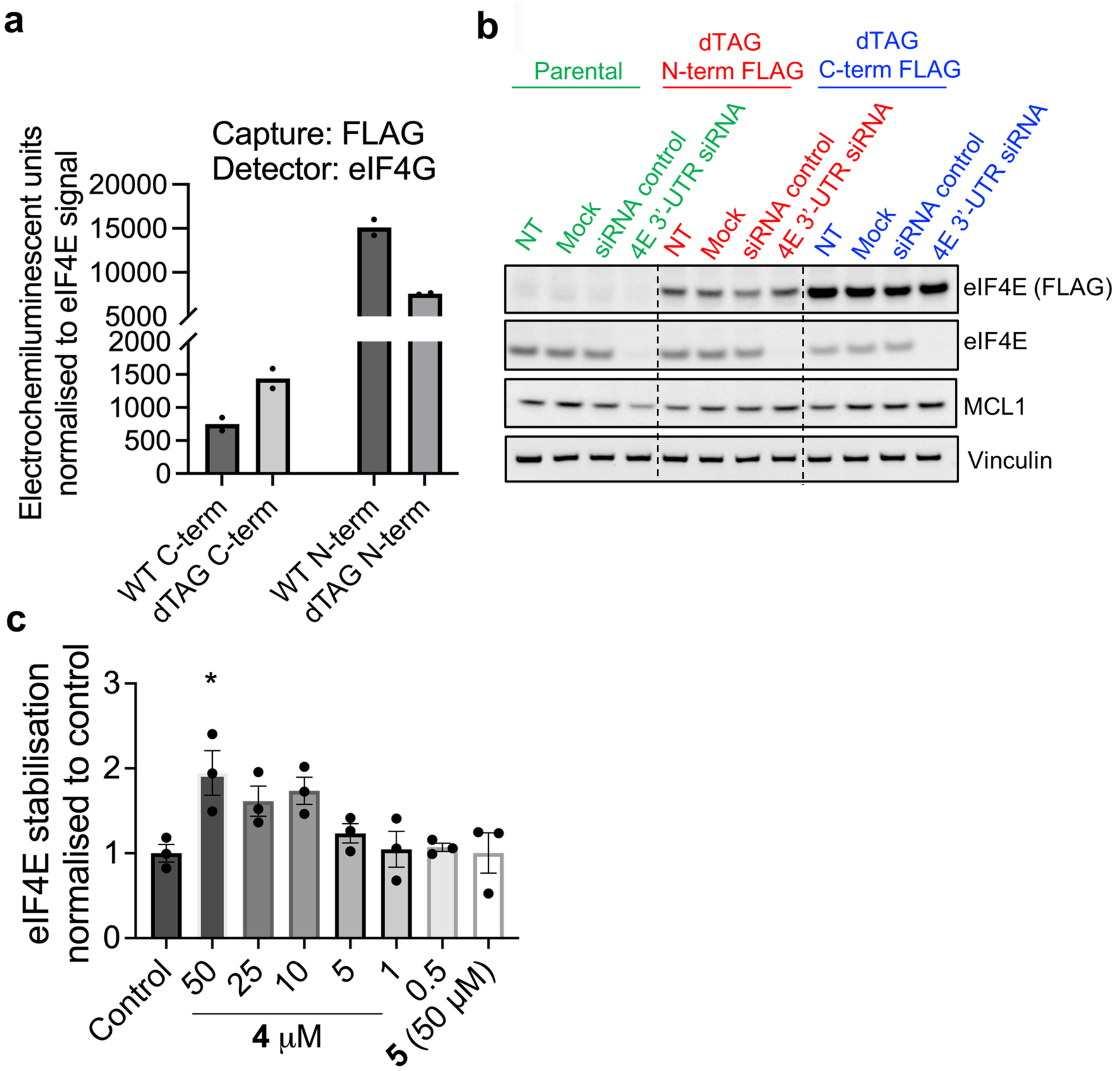

Supplementary Fig. 13 Quantification of the eIF4E:eIF4G interaction. **a** Quantification of the eIF4E:eIF4G interaction in H1299 cells transfected with either C- or N-terminal wild type FLAG (WT) or dTAG FKBP12^{F36V} (dTAG) tagged eIF4E, as measured by the electro-chemiluminescent assay. Each data point represents the mean luminescence signals from 2 technical repeats. **b** Representative immunoblot from one experiment of eIF4E, FLAG-tagged eIF4E and MCL1 protein expression in H1299 cells transfected with eIF4E 3'-UTR siRNA. Controls used were non-transfected cells (NT), mock control (lipid, no plasmid), negative control siRNA or an siRNA targeting the 3'UTR of eIF4E. Vinculin was used as a loading control. **c** Thermal stabilisation of eIF4E-dTAG protein by compound **4** or **5** binding. Quantification of immunoblots from H1299 C3-1cells incubated at 57.6°C, following treatment with compound **4** (50, 25, 10, 5, 1, 0.5 μM) or compound **5** (50 μM) for 6 hr. Vinculin was

used for loading control. eIF4E stabilisation normalised to control was quantified and data plotted as mean ± SEM from n = 3 biological replicates. An ordinary one-way ANOVA corrected for Tukey's multiple comparisons was performed. Only the control vs 50 µM showed significance ($p<0.05$) with $p=0.0286$. p values are denoted as $^{*}p<0.05$, source data is located in the Supplementary Source Data file.

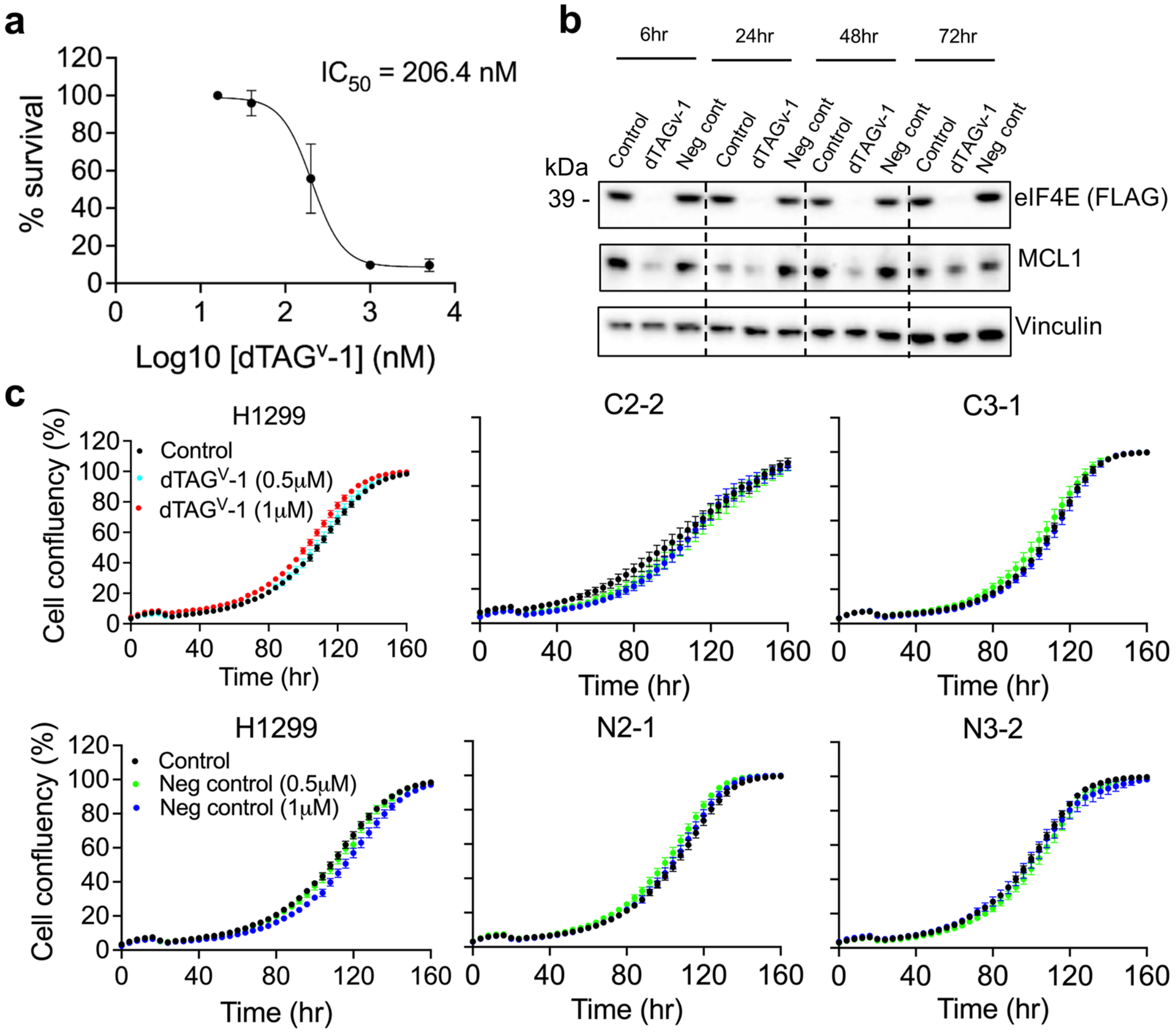

Supplementary Fig. 14 Treatment of the eIF4E-dTAG-degradation model with negative control dTAG^{V}-1 compound. a Plot of concentration responsiveness versus cell survival for H1299 clone C3-1 following 120 hr exposure to different concentrations of dTAG^{v}-1. Cell confluency (%) was calculated using Incucyte Zoom software and plotted as the mean ± SD from 3 biological replicates. **b** Immunoblot of eIF4E-dTAG (FLAG) and MCL1 protein expression in the eIF4E dTAG C3-1 single clone treated with 0.5 μM dTAG^{V}-1 or the negative control dTAG^{V}-1 compound for the indicated times. Vinculin was used as a loading control. The immunoblot shown is representative of 3 biological replicates. **c** Real-time cell confluency measurements of H1299 parental cells treated with 0.5 or 1 μM of dTAG^{V}-1 or the negative control dTAG^{V}-1 compound, and four selected eIF4E dTAG clones (C2-2, C3-1, N2-1 and N3-2) treated with 0.5 or 1 μM of the negative control dTAG^{V}-1 compound. Cells were monitored every 4 hr over a 6-day period. Cell confluency (%) was calculated using Incucyte Zoom software and shown as the mean ± SEM from 3 biological replicates.

An ordinary one-way ANOVA with Tukey multiple comparisons test found no significant differences ($p>0.05$) from controls, source data is located in the Supplementary Source Data file.

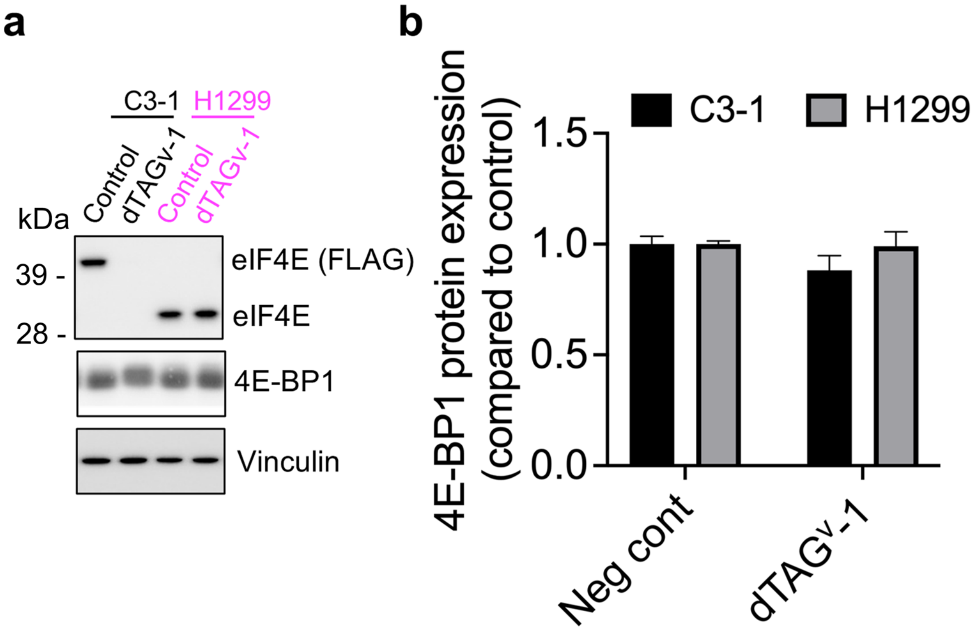

Supplementary Fig. 15 Expression of 4E-BP1 following dTAG^{V}-1 treatment. a Immunoblot of eIF4E, eIF4E-dTAG (FLAG) and 4E-BP1 protein expression following 16 hr treatment of 1 μM dTAG^{V}-1 or DMSO control in H1299 parental cells and the eIF4E dTAG C3-1 clone. Vinculin was used as a loading control. Results are representative of 3 biological replicates. **b** Quantification of 4E-BP1 protein expression in H1299 cells or the eIF4E dTAG C3-1 clone treated with 1 μM dTAG^{V}-1. Expression was normalised to the DMSO negative control immunoblot (mean ± SEM of 3 biological replicates). An unpaired t-test (two-tailed) did not detect any significance differences ($p<0.05$). Source data is located in the Supplementary Source Data file.

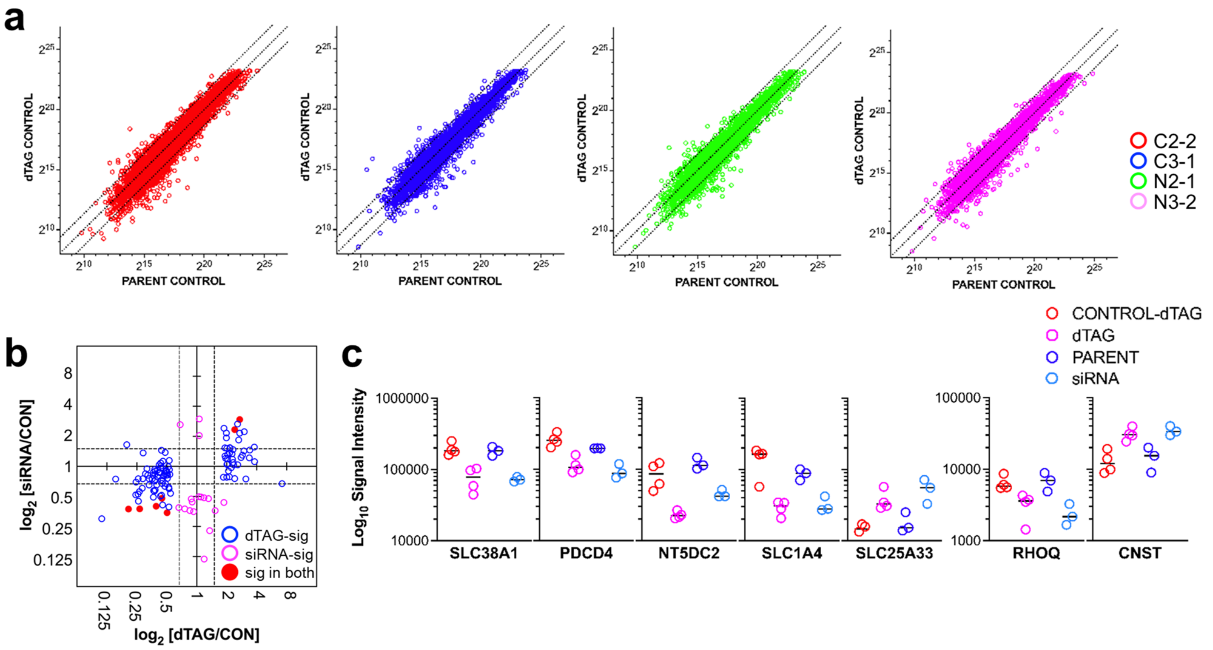

Supplementary Fig. 16 Comparison on eIF4E siRNA knockdown versus eIF4E-dTAG degradation. Parent H1299 cells were treated with 1 µg eIF4E or control siTOOLs for 72 hr (n= 3 biological replicates). The four eIF4E-dTAG clones (C2-2, C3-1, N2-1, N3-2) characterised in Fig. 7a-c were treated with 500 nM dTAG^{V}-1 for 72 hr (n = 1 independent repeat from each of the 4 individual clones). Total protein was extracted and global proteome profiled using data-independent acquisition (Supplementary Data 5). **a** Plots of signal intensity showing a comparison of the global proteome profiles of each individual dTAG clone with the control parent H1299 cells. **b** Plot of fold change for parent cells eIF4E siRNA/control siRNA versus eIF4E-dTAG clones dTAG^{V}-1 treated/DMSO control for proteins showing a significant difference (p_{adj} < 0.05; 1.5-fold change) from their control determined by MSstats (siRNA – 5 increased, 20 decreased; dTAG – 42 increased, 72 decreased). Eight proteins (EIF4E, RHOQ, SLC1A4, NT5DC2, SLC38A1, PDCD4, CNST and SLC25A33; filled red symbols) were significant in both siRNA and dTAG experiments and one third of proteins significantly (p_{adj} < 0.05; 1.5-fold change) altered in the dTAG experiment were also altered by 1.5-fold in the siRNA experiment (p_{adj}>0.05; not significant). **c** Signal intensity data for the 8 proteins showing significant difference (p_{adj} < 0,05; 1.5-fold change) between control and treatment for the siRNA and dTAG experiments. Each symbol represents data from an individual repeat.

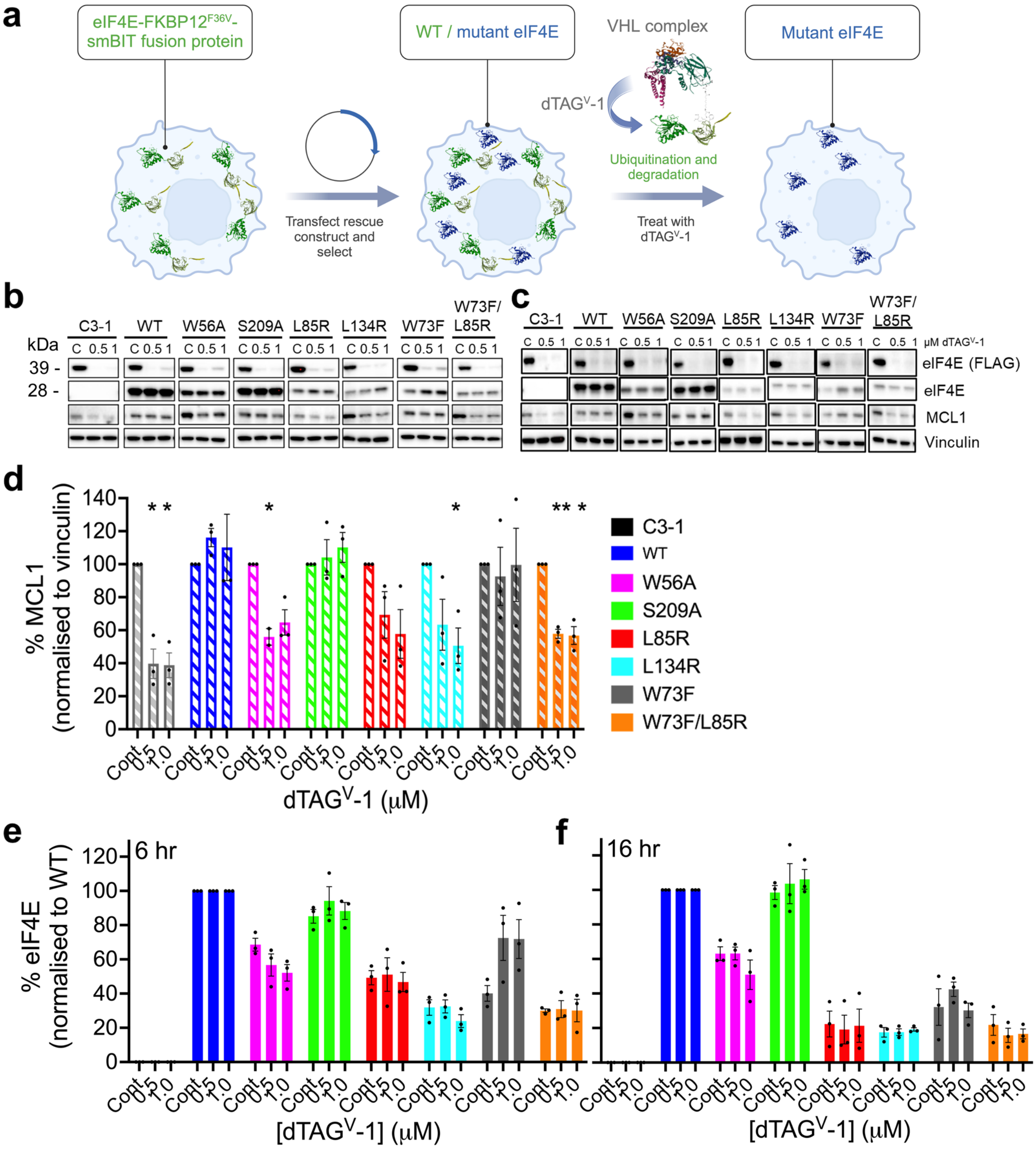

Supplementary Fig. 17. Transfection of wild type eIF4E or a series of mutants that impact on the different functional domains of eIF4E in the eIF4E dTAG C3-1 clone. a Scheme of the workflow to generate genetic rescue models by expression of wild type (WT) eIF4E or mutant eIF4E in the dTAG eIF4E CRISPR clones (Created in BioRender. Powers, M. (2024) https://BioRender.com/q02f176). **b** Representative immunoblot of eIF4E, FLAG-tagged eIF4E and MCL-1 protein expression following 6 hr or **c** 16 hr treatment of 0.5 or 1µM dTAG^{V}-1 in the eIF4E dTAG C3-1 clone transfected with wild-type eIF4E or a series of eIF4E mutants (n = 3 biological replicates). Vinculin was used as a loading control. The immunoblots were cropped to

aid interpretation of the data, original immunoblot images for all repeats are available in the Supplementary Source Data. **d** Quantification of MCL1 immunoblot signals using Image J. Results are expressed as % MCL-1 normalized to vinculin (mean ± SEM from (**c**) plus 2 additional biological replicates (see Supplementary Source Data). Significance ($p<0.05$) was determined using a one sample t-test (two-tailed) comparing treatment with the control from each cell line. Significance was observed for the C3-1 baseline cells (0.5 μM – p=0.0211; 1 μM p=0.0146), W56A (1μM – p=0.0444), L134R (1μM – p=0.0443) and W73F/L85R (0.5 μM – p=0.004; 1 μM p=0.0153) expressing cells. p values are denoted as *p<0.05, **p<0.01, ***p<0.001, ****p<0.0001. **e** Quantification of eIF4E normalised to WT expression (6 hr) from (**b**) and **f** 16 hr from (**c**), using Image J. A one sample t-test was performed for each cell treatment compared to their control and found no significant differences for either time point (p>0.05). Comparison of the control conditions for expression of each mutant with the overexpressed WT using an ordinary one-way ANOVA with Tukey multiple comparisons test found all mutants p<0.001 except S209A (p=0.0337) at 6 hr and all mutants except p<0.001except W56A (p=0.014) and S209 (p>0.05) at 16 hr.

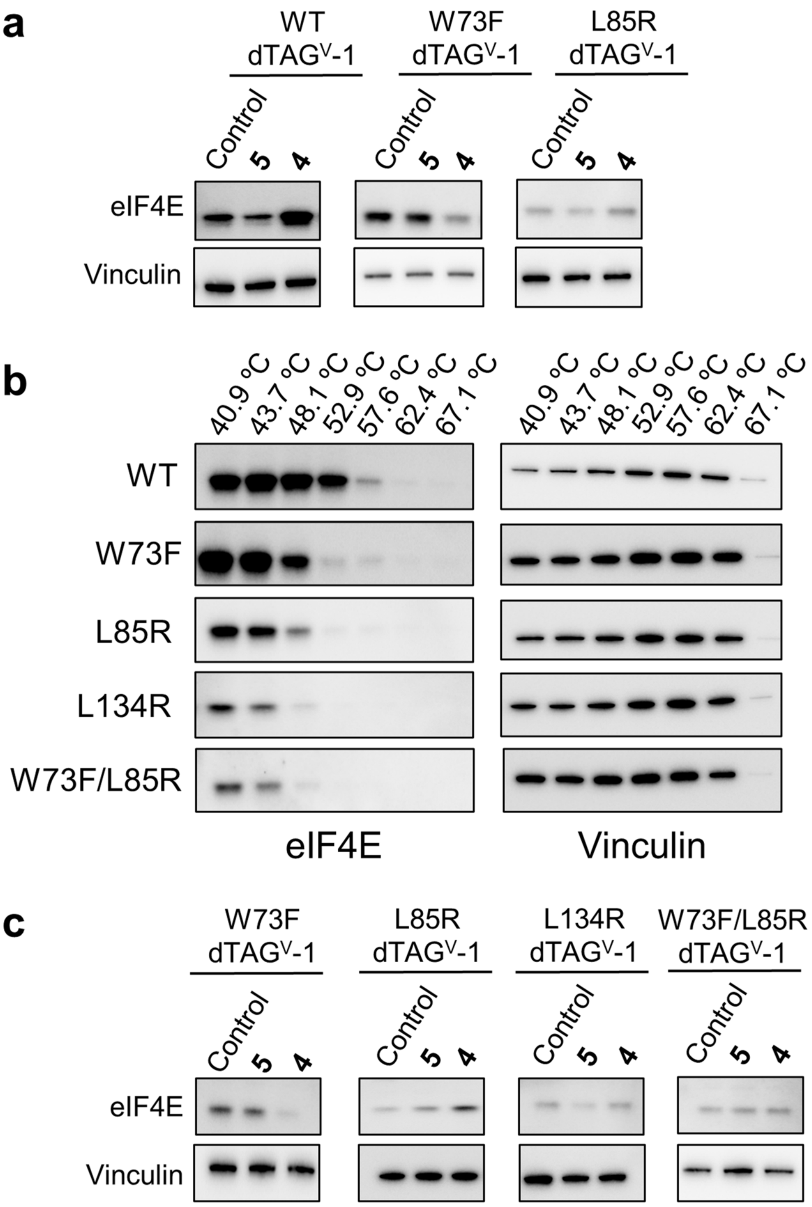

Supplementary Fig. 18. Comparison of compound 4 binding to wild-type or mutant eIF4E determined by thermal stabilisation in intact cells. a Immunoblots of eIF4E protein stabilisation at different temperatures in the eIF4E-dTAG model expressing wild-type, W73F or L85R eIF4E. Cells were treated with dTAGV-1 (1 μM) for 16 hr. Compound 4 (10 μM) or compound 5 (10 μM) was then added for 6 hrs. Vinculin was used for loading control. Blots are from one biological repeat. **b** Immunoblots showing eIF4E protein stabilisation at different temperatures. Vinculin was used as loading control. **c** Cells were treated with dTAGV-1 (1 μM) for 16 hr. Compound **4** (50 μM) or compound **5** (50 μM) was then added for 6 hrs. Immunoblots for eIF4E wild-type, W73F, L85R, L134R or W73F/L85R eIF4E mutants following incubation at 48.1 ^{o}C. Vinculin was used for loaing control. All blots are

from one biological repeat following up preliminary experiments defining the temperature range. Source data is located in the Supplementary Source Data file.

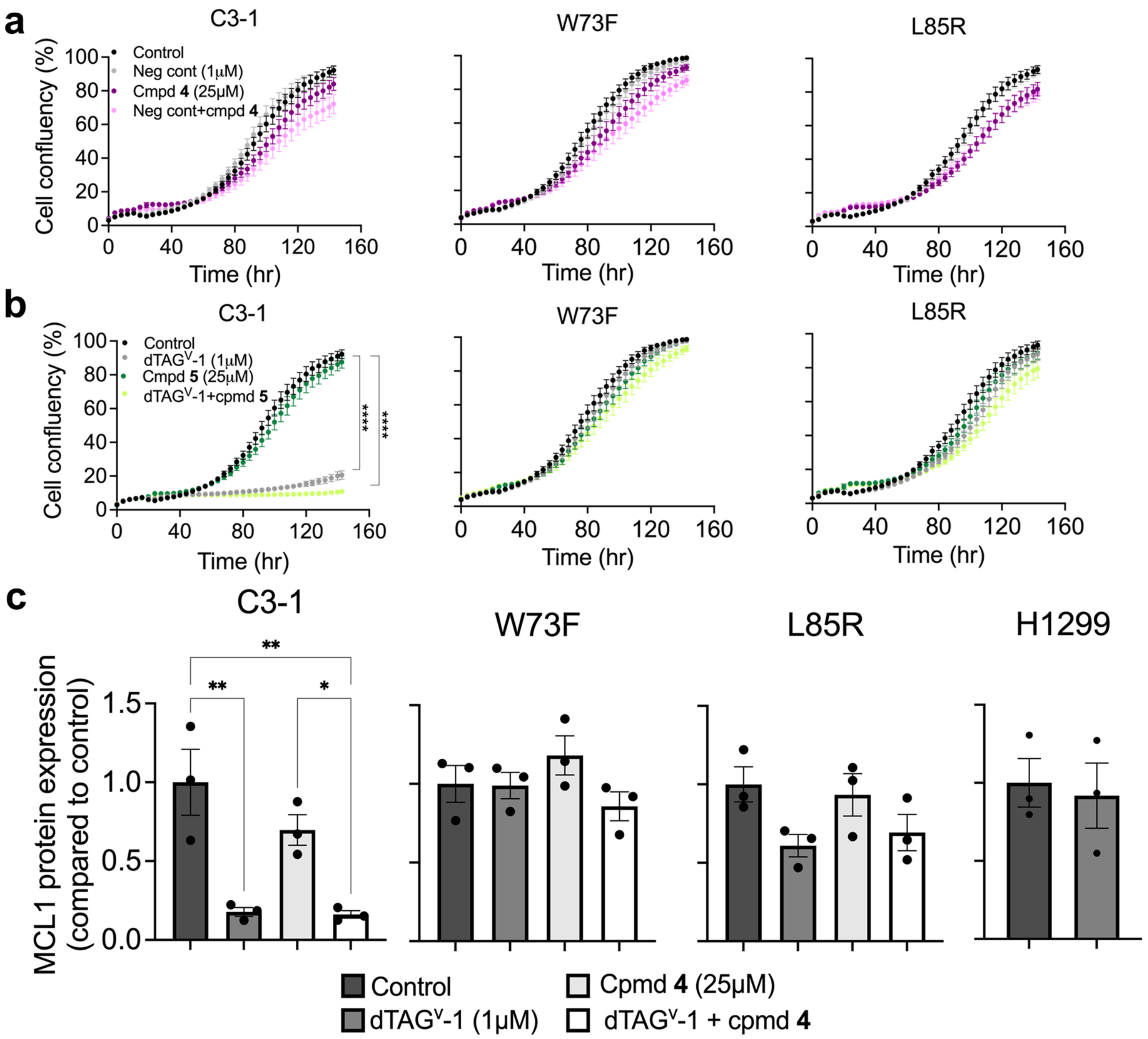

Supplementary Fig. 19 Determination of the effects of negative control dTAG^{V}-1 and compound 4, and dTAG^{V}-1 on the less active compound 5 on cell growth and MCL1 expression. a Real-time cell growth of the eIF4E-dTAG C3-1 clone and C3-1 transfected with W73F or L85R mutant following treatment with 25 μM compound **4** ± 1 μM negative control dTAG^{V}-1 or **b** 25 μM compound **5** ± dTAG^{V}-1 were monitored every 4 hr over a 6-day period. Cell confluency (%) was calculated using Incucyte Zoom software and shown as the mean ± SEM from 3 biological replicates. An ordinary one-way ANOVA was performed and compared with the control of each cell line or compound **4** or **5** alone. p values for all comparisons were insignificant (p>0.05) except for control vs control dTAG^{V}-1 (p<0.0001), dTAG^{V}-1 vs compound **5** (p<0.0001), in the C3-1 clone. **c** Quantification of MCL1 and vinculin immunoblot signals (from Fig. 8d) by Image J. Cell lysates from H1299 parental cells, eIF4E-dTAG C3-1 clone and C3-1 transfected with W73F or L85R mutant were treated with dTAG^{V}-1 (1 μM) or

compound **4 (**25 μM) or in combination for 16 hr. Results show protein expression levels normalised to vinculin and compared to their respective controls (mean ± SEM from 3 biological replicates). An ordinary one-way ANOVA was performed and compared with the control of each cell line, in the C3-1 line significant p values for control v dTAG (p=0.0047), control v dTAG + **4** (p=0.0042) and **4** v dTAG + **4** (p=0.0469). Values for all other comparisons were insignificant (p>0.05). p values are denoted as *p<0.05, **p<0.01, ***p<0.001, ****p<0.0001 and the source data is located in the Supplementary Source Data file.

2. SUPPLEMENTARY TABLES AND LEGENDS

	8QM4 (Cmpd 1)	8QM5 (Cmpd 2)	8QM6 (Cmpd 3)
**Data collection**			
Space group	*P*2*₁*	*P*2*₁*	*P*2*₁*
Cell dimensions			
a, *b*, *c* (Å)	47.15, 69.43, 63.82	47.12, 68.88, 63.73	47.29, 69.34, 64.77
α, β, γ (°)	90, 95.92, 90	90, 96.57, 90	90, 96.13, 90
Resolution (Å)	69.48-1.85(1.95-1.85)	38.72-1.89(1.96-1.89)	47.19-1.93(1.97-1.93)
R_{sym} or R_{merge}	0.048(0.714)	0.050(0.680)	0.051(0.916)
I / σ*I*	(2.3)	(1.9)	(1.5)
Completeness (%)	98.9(99.2)	98.4(99.1)	98.7(94.3)
Redundancy	3.3	3.3	2.9
**Refinement**			
Resolution (Å)	63.48-1.85	38.72-1.89	47.19-1.93
No. reflections	34696	32050	31021
R_{work} / R_{free}	0.193/0.217	0.194/0.220	0.187/0.227
No. atoms			
Protein	3299	3327	3309
Ligand	26	30	64
Water	120	191	260
B-factors			
Protein	47.5	45.7	39.4
Ligand	30.4	44.6	27.3
Water	49.6	50.9	45.8
R.m.s. deviations			
Bond lengths (Å)	0.13	0.13	0.12
Bond angles (°)	1.07	1.04	1.02

*Values in parentheses are for highest-resolution shell.

	8QM7 (Cmpd 5)	8QM9 (Cmpd 4)	8QM8 (Cmpd 4)
**Data collection**			
Space group	*P*2*₁*	*P*2*₁*	*P1*
Cell dimensions			
a, *b*, *c* (Å)	47.16, 69.12, 64.44	47.28, 69.27, 64.66	40.42, 42.03, 64.73
α, β, γ (°)	90, 95.67, 90	90, 95.71, 90	90.00, 90.01, 117.22
Resolution (Å)	64.12-2.19(2.27-2.19)	64.33-1.97(1.98-1.97)	64.73-1.58(1.63-1.58)
R_{sym} or R_{merge}	0.190(1.889)	0.122(1.662)	0.060(1.155)
I / σ*I*	(1.5)	(1.4)	(1.1)
Completeness (%)	95.9(93.5)	92.9(99.4)	70.6(6.6)
Redundancy	6.7	6.5	3.6
**Refinement**			
Resolution (Å)	63.48-1.85	47.05-1.97	64.73-1.585
No. reflections	34696	27229	36072
R_{work} / R_{free}	0.193/0.217	0.227/0.272	0.165/0.202
No. atoms			
Protein	3299	3309	3344
Ligand	50	50	50
Water	120	286	401
B-factors			
Protein	47.5	40.5	22.7
Ligand	30.4	28.6	16.5
Water	49.6	43.0	34.8
R.m.s. deviations			
Bond lengths (Å)	0.13	0.12	0.14
Bond angles (°)	1.07	1.04	1.21

*Values in parentheses are for highest-resolution shell.

Supplementary Table 1 Data collection and refinement statistics.

Mutation	Location	Reported impact	Predicted impact
L45F	Site 2		Disrupt site 2 ligand binding
W56A	Cap binding site	Prevent m7G Cap-binding[1]	Reported to prevent m7G Cap-binding
W73F	Canonical binding site	Cannot bind eIF4G or form an activated eIF4F complex[1]	
Y76A	Site 2	Maintain wild type function[2]	Disrupt site 2 ligand binding
I79F	Site 2		Affect conformation and/or disrupt site 2 ligand binding
L85R	Site 2/Flexible loop		Affect conformation
W130F	Site 2	Maintain wild type function[2]	Disrupt site 2 ligand binding
L134R	Site 2		Affect conformation and/or disrupt site 2 ligand binding
L134A	Site 2		Affect conformation and/or disrupt site 2 ligand binding
L134W	Site 2		Affect conformation and/or disrupt site 2 ligand binding
L134D	Site 2		Affect conformation and/or disrupt site 2 ligand binding
S209A	C-terminus	Cannot be phosphorylated or activated by MNK1/2[1, 3]	

Supplementary Table 2 Locations and reported or expected impact of eIF4E mutations used in binding and rescue studies. Some mutants were destabilising and poorly expressed (Supplementary Fig. 12c). W56A, W73F, L8R, L134R and S209A retained sufficient expression and were used for for followup studies.

3. SUPPLEMENTARY NOTES: COMPOUND SYNTHETIC METHODS, INCLUDING ANALYTICAL CHARACTERISATION AND EXPERIMENTAL PROCEDURES FOR THE MEASUREMENT OF CHROMLOGD, AQUEOUS SOLUBILITY, PLASMA PROTEIN BINDING, CACO2 PERMEABILITY AND MICROSOMAL CLEARANCE.

General chemistry. All solvents and commercially available reagents were used as received. All reactions were followed by TLC analysis (TLC plates GF254, Merck) or LC−MS (liquid chromatography mass spectrometry). Column chromatography was performed on prepacked silica gel columns (3090 mesh, IST) using a Biotage SP4 or similar. NMR spectra were measured on a Bruker 400MHz AVIII HD Nanobay using the parameters included in table below, processed using MestReNova software (version 14.3.1) and are referenced as follows: ^{1}H (400 MHz), internal standard TMS at δ = 0.00. Abbreviations for multiplicities observed in NMR spectra: s; singlet; br s, broad singlet; d, doublet; t, triplet; q, quadruplet; p, pentuplet; spt, septuplet; m, multiplet. UPLC-UV-MS analysis was performed with an Agilent or Shimadzu UHPLC system with variable wavelength UV detection using reverse-phase chromatography with a CH_3CN and water gradient with modifier (added to each solvent) and using a reverse-phase column, for example, Thermo Hypersil Gold C18 or Waters Acquity UHPLC BEH C18. Modifiers were either "basic" 10 mM ammonium bicarbonate + NH_4OH (pH 9.4) or "acidic" 0.1% formic acid (pH 3.0). MS was determined using either PE Sciex 150EX LC−MS, Waters ZQLC−MS, or Agilent 6140 LC−MS Single Quadrupole instruments. High resolution mass spectra (HRMS) was determined using a Waters Acquity I Class UPLC / Waters IMS Q-TOF Mass Spectrometer using ESI (electrospray ionisation). All compounds reported are of at least 95% purity according to LC−MS unless otherwise stated.

Compound **1** ([1,1'-biphenyl]-3-ol) and **2** (1-(4-chlorophenyl)cyclopentane-1-carboxylic acid) were purchased from commercial sources (Sigma Aldrich and Acros respectively). Compounds **3-5** were prepared as follows:

Compound 3: 1-{2'-fluoro-[1,1'-biphenyl]-4-yl}ethan-1-ol

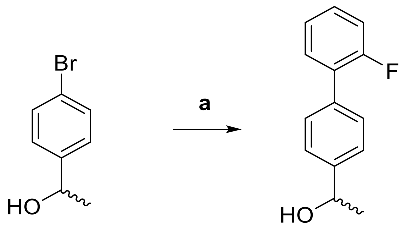

(a) 2-Fluorophenyl-$B(OH)_2$, $Pd(dppf)Cl_2$, Na_2CO_3, 1,4-dioxane / H_2O, 120 ºC

(a) A solution of 4-bromo-α-methylbenzylalcohol (300mg; 1.5mmol), 2-fluorophenylboronic acid (315mg; 3mmol) and [1,1′-bis(diphenylphosphino)ferrocene]dichloropalladium(II) (120mg; 10 mol%), 1M sodium carbonate (4.5ml; 4.5mmol) in 1,4-dioxane (9ml) was heated at 120°C for 30 minutes under microwave irradiation. The reaction was cooled and partitioned between ethyl acetate and water. The ethyl acetate layer was separated, dried over sodium sulfate, filtered, and evaporated. The crude material was purified by SiO_2 flash column chromatography using gradient elution from with 0% to 25% ethyl acetate / petroleum ether. Product containing fractions were combined and evaporated to give 260mg (80%) of 1-{2'-fluoro-[1,1'-biphenyl]-4-yl}ethan-1-ol as a white solid. ^1H NMR (400 MHz, DMSO-d6): 7.56-7.36 (m, 6H), 7.36-7.19 (m, 2H), 5.18 (d, J = 4.1 Hz, 1H), 4.83-4.73 (m, 1H), 1.37 (d, J = 6.5 Hz, 3H).

Compound 4: (2R)-2-[(1S)-1-{2'-fluoro-3-[(2-hydroxyethyl)amino]-[1,1'-biphenyl]-4-yl}propoxy]propan-1-ol

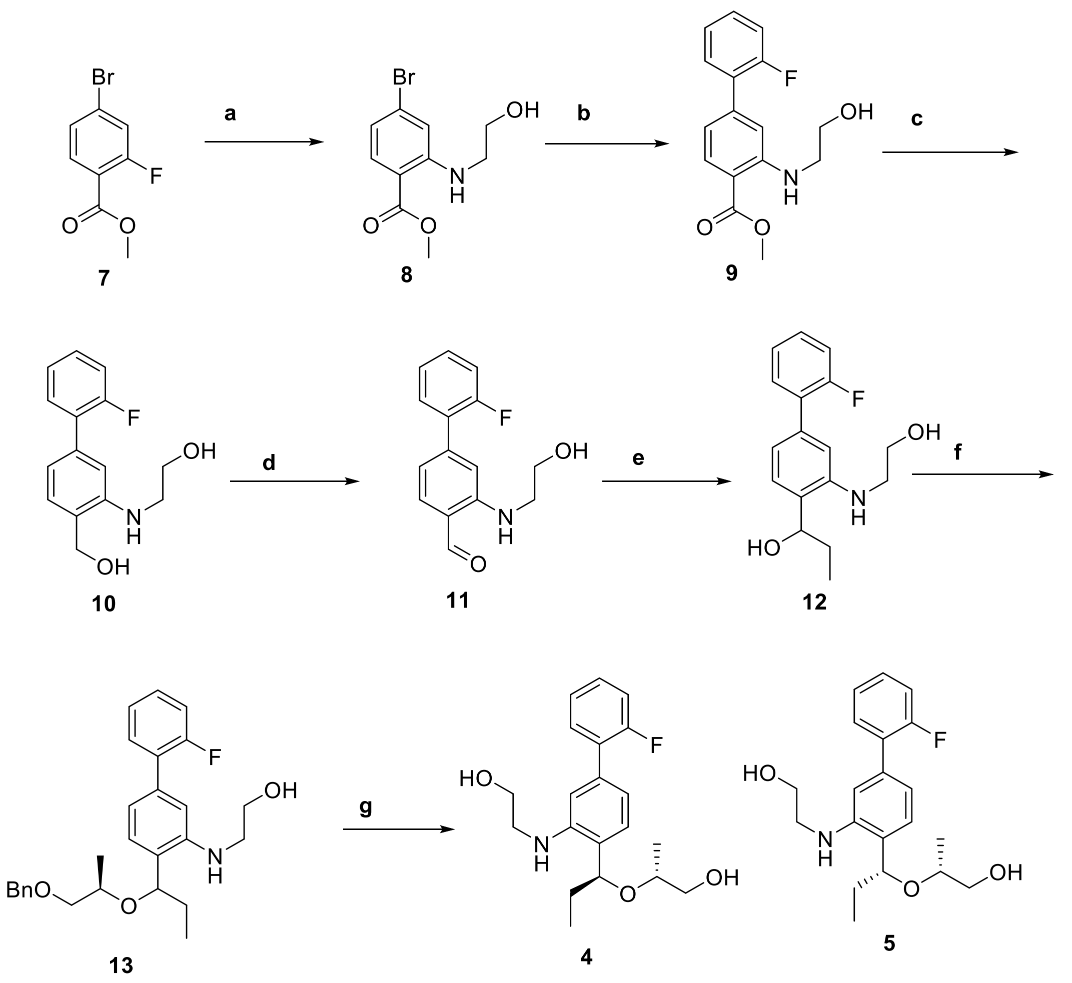

Reagents and conditions: (a) $H_2N(CH_2)_2OH$, NEt_3, NMP, 100°C; (b) 2-Fluorophenyl-$B(OH)_2$, $Pd(dtbpf)Cl_2$, K_2CO_3, MeCN / H_2O, 80 ºC; (c) $LiAlH_4$, THF, 0 ºC; (d) MnO_2, DCM; (e) EtMgBr, THF, 0 ºC-rt; (f) (R)-$BnOCH_2CH(CH_3)OH$, 100 ºC; 10% Pd/C, H_2, MeOH

Step 1 (a): A mixture of methyl 2-fluoro-4-bromobenzoate (85.0g; 365mmol), ethanolamine (33ml; 547mmol) and triethylamine (5.1ml; 365mmol) in NMP (425ml) was heated to 100°C for 16hr. The reaction was cooled to room temperature, water (1275ml) was added. Mixture was stirred until a thick precipitate had formed. The precipitate was collected by filtration, washed with water (200ml) and petrol (200ml) and dried at 40C overnight to give 78.6g (78%) of methyl 4-bromo-2-[(2-hydroxyethyl)amino]benzoate. NMR ^1H NMR (400 MHz, $CDCl_3$) δ 8.01 (s, 1H), 7.77 (d, J = 8.5 Hz, 1H), 6.90 (d, J = 1.9 Hz, 1H), 6.75 (dd, J = 8.5, 1.9 Hz, 1H), 3.91 (t, J = 5.4 Hz, 3H), 3.87 (s, 3H), 3.41 (q, J = 5.4 Hz, 3H), 1.71 (s, 2H), 1.61 – 1.56 (m, 1H).

Step 2 (b): A mixture of methyl 4-bromo-2-[(2-hydroxyethyl)amino]benzoate (14.8g; 54mmol), 2-fluorophenylboronic acid (8.3g; 59.4mmol), [1,1′-Bis(di-tert-butylphosphino)ferrocene]dichloropalladium(II) (0.176g; 0.27mmol) and potassium carbonate (11.2g; 81mmol) in MeCN/H_2O (1:1; 118ml) was heated to 80°C for 1hr. After cooling to room temperature the phases were separated and the aqueous phase was extracted with ethyl acetate (2 x 75ml). The combined organic phases were washed with brine (75ml), dried over $MgSO_4$ and evaporated to give 15.2g (97%) of methyl 2'-fluoro-3-[(2-hydroxyethyl)amino]-[1,1'-biphenyl]-4-carboxylate. ^{1}H NMR (400 MHz, $CDCl_3$) δ 8.00 (d, *J* = 8.3 Hz, 1H), 7.48 (td, *J* = 7.7, 1.8 Hz, 1H), 7.37 (dddd, *J* = 8.2, 7.4, 5.0, 1.8 Hz, 1H), 7.24 (td, *J* = 7.5, 1.2 Hz, 1H), 7.18 (ddd, *J* = 10.8, 8.2, 1.2 Hz, 1H), 6.93 (t, *J* = 1.6 Hz, 1H), 6.82 (dt, *J* = 8.3, 1.7 Hz, 1H), 3.91 (s, 5H), 3.49 (q, *J* = 5.5 Hz, 2H), 1.72 (s, 1H).

Step 3 (c): A solution of methyl 2'-fluoro-3-[(2-hydroxyethyl)amino]-[1,1'-biphenyl]-4-carboxylate (15.1g; 52.2mmol) in anhydrous THF (75ml) was added slowly to a stirring suspension of lithium aluminium hydride (1.98g; 52.2mmol) in anhydrous THF (150ml) at 0°C under N_2. Reaction was stirred at 0C for 1.5hr before a further addition of lithium aluminium hydride (0.495g; 13.0mmol) was added and the reaction was stirred for a further 0.5hr. The reaction was quenched by sequential addition of water (2.5ml), 2M NaOH (2.5ml) and water (7.5ml). The reaction mixture was warmed to room temperature and stirred for an hour before being filtered to remove the precipitate. The precipitate was washed with ethyl acetate (50ml) and the filtrate was evaporated to give 14.8g (108%) of 2-{[2'-fluoro-4-(hydroxymethyl)-[1,1'-biphenyl]-3-yl]amino}ethan-1-ol. ^{1}H NMR (400 MHz, $CDCl_3$) δ 7.45 (td, *J* = 7.7, 1.9 Hz, 1H), 7.35 – 7.29 (m, 1H), 7.21 (td, *J* = 7.5, 1.3 Hz, 1H), 7.19 – 7.13 (m, 2H), 6.92 – 6.85 (m, 2H), 3.88 (dd, *J* = 5.6, 4.8 Hz, 2H), 3.41 (t, *J* = 5.2 Hz, 2H).

Step 4 (d): Manganese dioxide (45.4g; 522mmol) was added to a stirring solution of 2-{[2'-fluoro-4-(hydroxymethyl)-[1,1'-biphenyl]-3-yl]amino}ethan-1-ol (13.6g; 52.2mmol) in DCM (273ml) and the reaction was stirred for 18hr. The mixture was filtered through GF/A glass microfibre filter paper and the filtrate was evaporated to give 12.05g (89%) of 2'-fluoro-3-[(2-hydroxyethyl)amino]-[1,1'-biphenyl]-4-carboxaldehyde. ^{1}H NMR (400 MHz, $CDCl_3$) δ 9.90 (d, *J* = 0.7 Hz, 1H), 8.56 (s, 1H),

7.57 (d, *J* = 7.9 Hz, 1H), 7.48 (td, *J* = 7.7, 1.8 Hz, 1H), 7.39 (dddd, *J* = 8.1, 7.0, 5.0, 1.8 Hz, 1H), 7.28 – 7.15 (m, 3H), 6.99 – 6.88 (m, 2H), 3.93 (q, *J* = 5.5 Hz, 2H), 3.52 (q, *J* = 5.6 Hz, 2H), 1.77 (t, *J* = 5.7 Hz, 1H).

Step 5 (e): A 2M solution of ethylmagnesium chloride in dry THF (21ml; 42mmol) was added dropwise to a solution of 2'-fluoro-3-[(2-hydroxyethyl)amino]-[1,1'-biphenyl]-4-carboxaldehyde (3.1g; 12mmol) in anhydrous THF at 0°C, stirred cold for 30 minutes then allowed to warm to room temperature. The reaction was quenched with saturated ammonium chloride solution, diluted with brine and extracted with ethyl acetate. The ethyl acetate layer was separated, dried over sodium sulphate, filtered, and evaporated. The crude material was purified by SiO_2 flash column chromatography using gradient elution from with 0% to 100% ethyl acetate / petroleum ether. Product containing fractions were combined and evaporated to give 1.2g (35%) of 1-{2'-fluoro-3-[(2-hydroxyethyl)amino]-[1,1'-biphenyl]-4-yl}propan-1-ol as a yellow solid. MS: $[M+H]^+$ = 290. ^1H NMR (400 MHz, DMSO-d6): 7.54-7.47 (1H, m), 7.42-7.34 (1H, m), 7.31-7.23 (2H, m), 7.11 (1H, d), 6.75-6.67 (2H, m), 5.61 (1H, t), 5.31 (1H, d), 4.73 (1H, t), 4.57-4.48 (1H, m), 3.68-3.55 (2H, m), 3.22-3.10 (2H, m), 1.83-1.66 (2H, m), 0.88 (3H, t).

Step 6 (f): A mixture of 1-{2'-fluoro-3-[(2-hydroxyethyl)amino]-[1,1'-biphenyl]-4-yl}propan-1-ol (480mg; 1.66mmol) and (R)-1-benzyloxy-2-propanol (1ml) was heated under microwave irradiation at 100 ºC for 90 minutes then purified by reverse phase flash column chromatography eluting with 40% to 100% acetonitrile / water. Product containing fractions were combined and evaporated to give 165mg (23%) of 2-{[4-(1-{[(2R)-1-(benzyloxy)propan-2-yl]oxy}propyl)-2'-fluoro-[1,1'-biphenyl]-3-yl]amino}ethan-1-ol as a pale yellow gum. MS: $[M+H]^+$ = 438.

Step 7 (g): A solution of 2-{[4-(1-{[(2R)-1-(benzyloxy)propan-2-yl]oxy}propyl)-2'-fluoro-[1,1'-biphenyl]-3-yl]amino}ethan-1-ol (200mg; 0.46mmol) in methanol (10ml) was treated with 10% palladium on carbon then hydrogenated at room temperature and pressure overnight. The reaction was filtered through celite, and the filtrate was evaporated. The crude material was purified by reverse phase preparative HPLC to give separate diastereoisomers.

Compound **4**: 20mg (12.5%) of (2R)-2-[(1S)-1-{2'-fluoro-3-[(2-hydroxyethyl)amino]-[1,1'-biphenyl]-4-yl}propoxy]propan-1-ol was isolated as a colourless gum. HRMS m/z: $[M+H]^+$ calculated for $C_{20}H_{26}FNO_3$ 348.1980; found 348.1974. ^1H NMR (400 MHz, DMSO-d6): 7.58-7.45 (1H, m), 7.45-7.33 (1H, m), 7.33-7.22 (2H, m), 7.11 (1H, d), 6.79-6.66 (2H, m), 5.68 (1H, t), 4.81 (1H, t), 4.58 (1H, t), 4.44 (1H, t), 3.69-3.55 (2H, m), 3.44-3.31 (2H, m), 3.28-3.08 (3H, m), 1.92-1.67 (2H, m), 1.08 (3H, d), 0.86 (3H, t).

Compound **5**: 30mg (19%) of (2R)-2-[(1R)-1-{2'-fluoro-3-[(2-hydroxyethyl)amino]-[1,1'-biphenyl]-4-yl}propoxy]propan-1-ol was isolated as a colourless gum. HRMS m/z: $[M+H]^+$ calculated for $C_{20}H_{26}FNO_3$ 348.1980; found 348.1973. ^1H NMR (400 MHz, DMSO-d6): 7.57-7.48 (1H, m), 7.43-7.33 (1H, m), 7.32-7.22 (2H, m), 7.08 (1H, d), 6.77-6.67 (2H, m), 5.59 (1H, t), 4.74 (1H, t), 4.55-4.45 (2H, m), 3.68-3.55 (2H, m), 3.55-3.44 (1H, m), 3.44-3.31 (2H, m), 3.24-3.08 (2H, m), 1.90-1.62 (2H, m), 1.02 (3H, d), 0.85 (3H, t).

^1H NMR Spectrum (400 MHz, DMSO-d_6) of compound 4

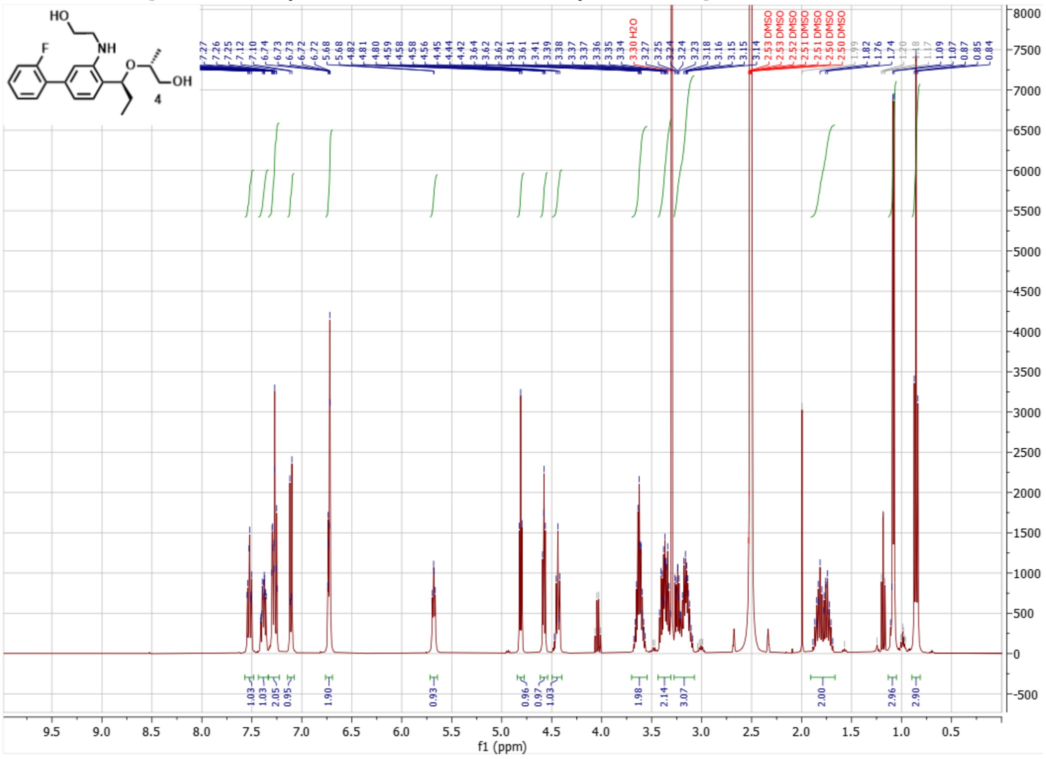

^1H NMR Spectrum (400 MHz, DMSO-d_6) of 5

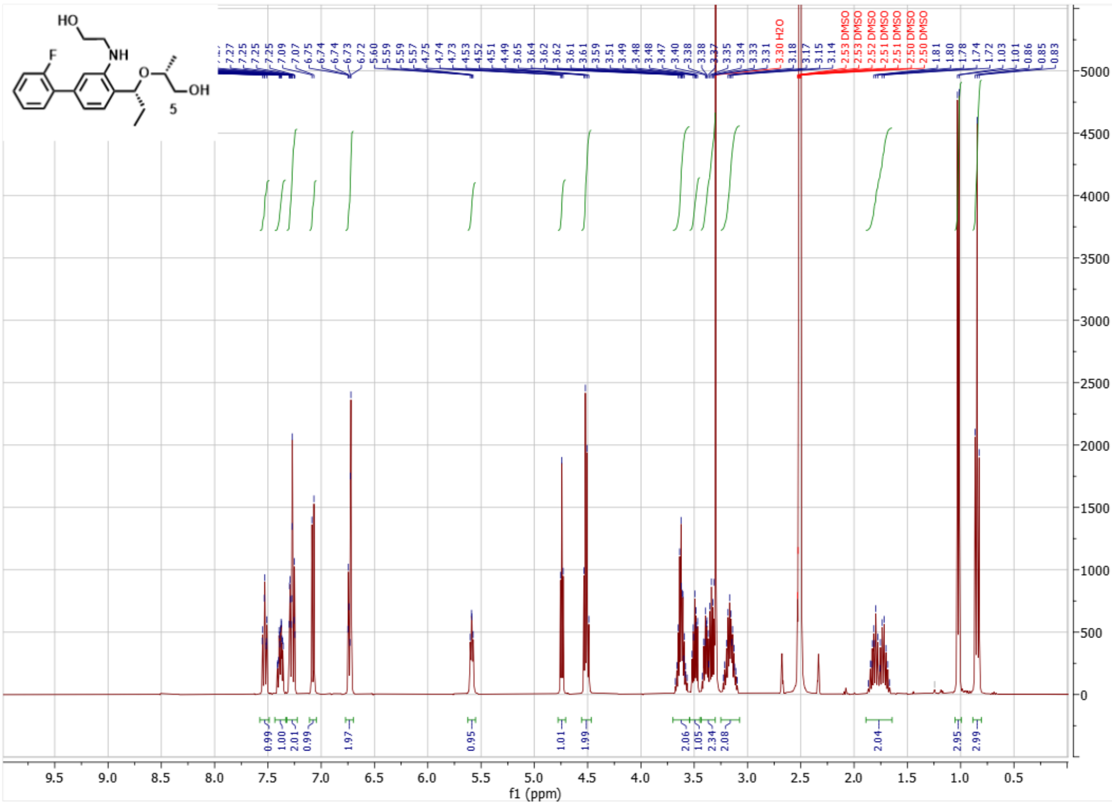

Overlaid ^{1}H NMR Spectrum (400 MHz, DMSO-d$_6$) of compounds 4 (red) and 5 (blue)

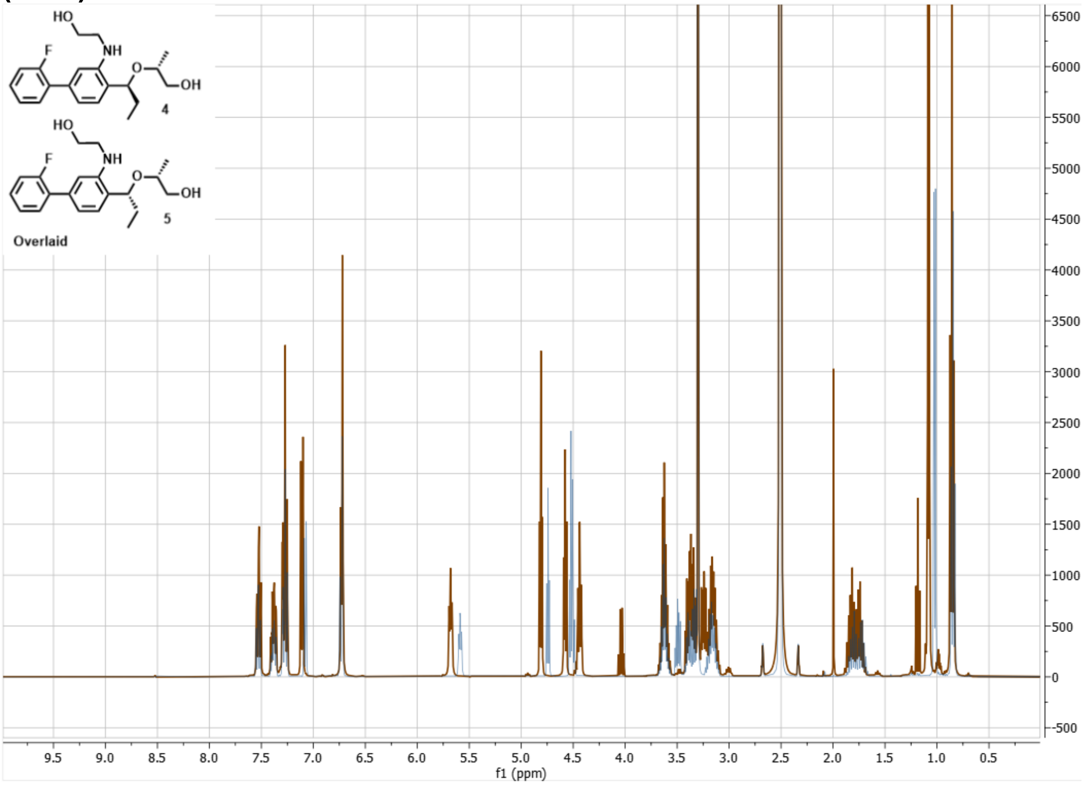

Supplementary notes table including NMR spectrometer parameters

Spectrometer	400MHz AVIII HD Nanobay
Probe	5mm BBO room temperature
Software	Topspin
Pulse sequence	zg30
Sweep width	20ppm (default)
Acquisition time	4.0894465 secs
Number of scans	16 (default)
Dummy scans	4 (default)
Temperature	300K (default)
P1 90 degree high power pulse	10 μsec
D1 relaxation delay	1 sec
Spinning rate	20Hz
Receiver gain	Will be optimised automatically per sample by ICON

High performance liquid chromatography purity data for compound 4

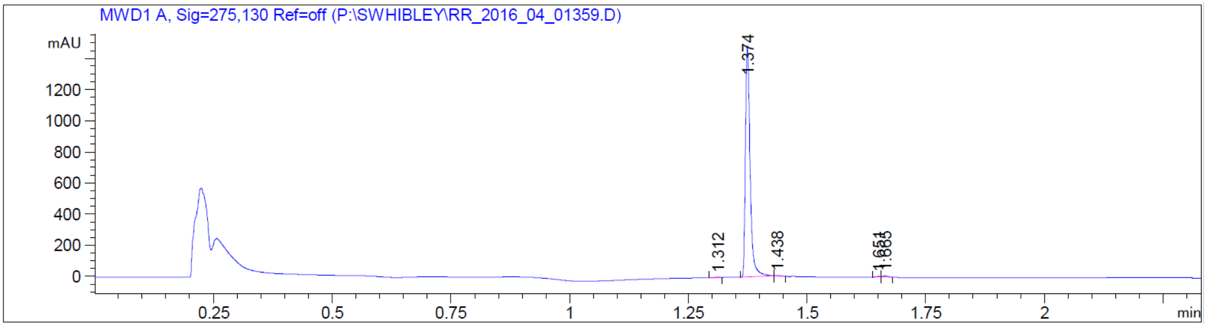

Peak #	RetTime [min]	Type	Width [min]	Area [mAU*s]	Height [mAU]	Area %
1	1.312	BB	9.48e-3	1.41921	2.21472	0.1381
2	1.374	BB	0.0105	1019.29749	1484.67285	99.1686
3	1.438	BB	8.71e-3	1.68376	3.04170	0.1638
4	1.651	BB	0.0101	1.59146	2.52530	0.1548
5	1.665	BB	0.0103	3.85144	5.93984	0.3747

High performance liquid chromatography purity data for compound 5

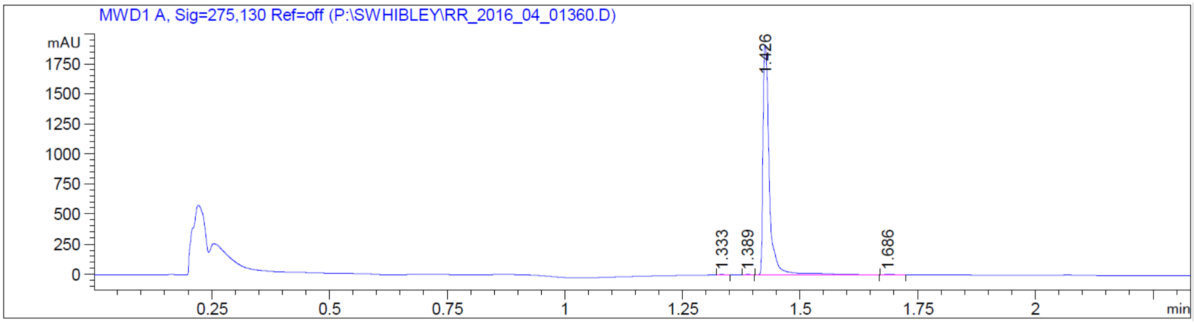

```
Peak RetTime Type  Width      Area       Height      Area
  #   [min]        [min]    [mAU*s]      [mAU]        %
----|-------|----|-------|----------|----------|--------|
   1   1.333 BB    0.0107    3.19010    4.56165   0.1758
   2   1.389 BB   9.11e-3    1.95645    3.32867   0.1078
   3   1.426 BB    0.0148 1805.41089 1906.89124  99.4821
   4   1.686 BB    0.0203    4.25222    2.86393   0.2343
```

**Cmpd #**	**Chemical Structure**	**MW (Da)**	**Chrom LogD (pH 7.5)**	**Aqueous kinetic solubility (pH7.5) (µM)**	**Plasma protein binding (%)**	**Caco-2 permeability (Papp x10^{-6} cm/s)**	**Human microsomal intrinsic clearance (µL/min/mg protein)**
**4**	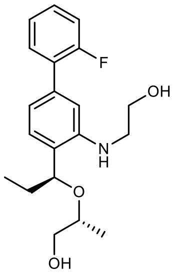	347.5	4.6	400	98.9	9.0	210

Supplementary notes Table Physicochemical and *in vitro* pharmacokinetic properties of compound 4.

Experimental procedures for the measurement of ChromLogD, aqueous solubility, plasma protein binding, Caco2 permeability and microsomal clearance.

Determination of Chromatographic LogD (ChromLogD$_{7.4}$)

Chromatographic hydrophobicity index (CHI) values were measured using reversed phase HPLC column (PolymerX RP-1, Phenomenex, UK) with a fast acetonitrile gradient at starting mobile phase of pH = 7.4. CHI values are derived directly from the gradient retention times by using a calibration line obtained for standard compounds. The CHI value approximates to the volume % organic concentration when the compound elutes. CHI is linearly transformed into ChromLogD$_{7.4}$ by least-square fitting of experimental CHI values to calculated C logP values for over 20,000 research compounds using the following formula: ChromLogD$_{7.4}$ = 0.0857CHI-2.00[1].

Measurement of aqueous kinetic solubility at pH 7.5

Solubility was measured in 50 mM HEPES, 100 mM NaCl (15% D2O, 85% H2O) in the presence of 1 mM TCEP at pH 7.5. Samples were prepared at 50 mM in DMSO-d6, diluted x 50 into buffer affording nominal concentrations of 1 mM and analysed by ^{1}H quantitative NMR. Spectra were acquired at 300K in 5 mm tubes on a 400 MHz Bruker Avance III HD spectrometer equipped with a BBO-F probe using water suppression (presat/Watergate). A 5 mM sample of 4-hydroxybenzoic acid was used as the qNMR standard. The raw data files were processed using MNova with qNMR plugin[2].

Caco-2 permeability Assay

The permeability of Compound 4 was assessed using the CacoReady system. Compound 4 and control compounds (propranolol, antipyrine, and vinblastine) were incubated at a final concentration of 10 µM in duplicate to either the apical (180 µL) of the monolayer to measure apical to basolateral transport (A > B) across the cell barrier or to the basolateral side (750 µL) to measure the basolateral to apical transport (B > A). For A > B spiking, test and control compounds were diluted from 10 mM DMSO stocks to 10 µM in HBSS buffer with 100 mM $CaCl_2{\cdot}2H_2O$, 50 mM $MgCl_2{\cdot}5H_2O$, and 0.5 mg/mL lucifer yellow made to volume with sterile water (lucifer yellow was used to determine the integrity of the Caco-2 monolayer). For B > A compound spiking, test and control compounds were prepared as for the A > B solutions without the addition of lucifer yellow. Test and control compounds were incubated for 1 h at +37 °C in a highly humidified atmosphere of 95% air and 5% CO_2.

Plasma Protein Binding Assay

Plasma protein binding measured using the rapid equilibrium dialysis (RED) device system. In duplicate, 300 µL of human plasma containing 1 µM compound 4 was added to the plasma cmpt of a red device and 500 µL of PBS adding to the surrounding environment following manufacturer guidelines. The red device was shaken at 120 rpm and at 37°C, 5% CO_2 for 16 hrs before samples were taken from the plasma and buffer cmpts. Percent bound was calculated as the ([plasma cmpt] / [plasma cmpt +

buffer cmpt]) * 100. The assay controls, ketoconazole, verapamil and carbamazepine were incubated under the same conditions.

Human microsomal clearance assay.

Compound **4** was incubated in duplicate at 1 µM in male CD-1 mouse liver microsomes (0.25 mg/mL) at +37 °C for 40 min in the presence of NADPH. The positive controls, diclofenac and midazolam, were incubated under the same conditions. Negative control incubations, in the absence of NADPH, were performed. Incubations were sampled (50 µL) at 0, 5, 10, 20, and 40 min, except for the negative controls, which were sampled at 0 and 40 min.

4. SUPPLEMENTARY REFERENCES

1. Valko, K., Bevan, C. & Reynolds, D. Chromatographic Hydrophobicity Index by Fast-Gradient RP-HPLC: A High-Throughput Alternative to log P/log D. *Anal Chem* **69**, 2022-2029 (1997).
2. Farrant, R.D. *et al.* NMR quantification using an artificial signal. *Magn Reson Chem* **48**, 753-762 (2010).
