# Supplementary material for: Integrating fragment-based screening with targeted protein degradation and genetic rescue to explore eIF4E function
Source: Nat Commun. 2024 Nov 20;15:10037. doi: 10.1038/s41467-024-54356-1 (PMC11868579; doi:10.1038/s41467-024-54356-1)
Supplement: Supplementary file 2 — Description of Additional Supplementary Files [file 41467_2024_54356_MOESM2_ESM.pdf]

Description of Additional Supplementary Files

File Name: Supplementary Data 1
Description: eIF4E protein constructs evaluated prior to fragment screening.

File Name: Supplementary Data 2
Description: Amino acid sequences of clones referred to in Supplementary Table 1.

File Name: Supplementary Data 3
Description: Sequence conservation comparison of representative eIF4E orthologs compared to human eIF4E.

File Name: Supplementary Data 4
Description: Sequence conservation comparison of the complete list of eIF4E orthologs compared to human eIF4E.

File Name: Supplementary Data 5
Description: Summary of proteome profiles analysed by DIA following eIF4E siRNA or dTAGV-1 treatment.
