# Supplementary material for: Integrating fragment-based screening with targeted protein degradation and genetic rescue to explore eIF4E function
Source: Nat Commun. 2024 Nov 20;15:10037. doi: 10.1038/s41467-024-54356-1 (PMC11868579; doi:10.1038/s41467-024-54356-1)
Supplement: Supplementary file 8 — Reporting Summary [file 41467_2024_54356_MOESM8_ESM.pdf]

nature portfolio

Corresponding author(s): PAUL ANDREW CLARKE

Last updated by author(s): 25/10/24

Reporting Summary

Nature Portfolio wishes to improve the reproducibility of the work that we publish. This form provides structure for consistency and transparency in reporting. For further information on Nature Portfolio policies, see our Editorial Policies and the Editorial Policy Checklist.

Statistics

For all statistical analyses, confirm that the following items are present in the figure legend, table legend, main text, or Methods section.

n/a	Confirmed	
☐	☒	The exact sample size (*n*) for each experimental group/condition, given as a discrete number and unit of measurement
☐	☒	A statement on whether measurements were taken from distinct samples or whether the same sample was measured repeatedly
☐	☒	The statistical test(s) used AND whether they are one- or two-sided<br>*Only common tests should be described solely by name; describe more complex techniques in the Methods section.*
☐	☒	A description of all covariates tested
☐	☒	A description of any assumptions or corrections, such as tests of normality and adjustment for multiple comparisons
☐	☒	A full description of the statistical parameters including central tendency (e.g. means) or other basic estimates (e.g. regression coefficient) AND variation (e.g. standard deviation) or associated estimates of uncertainty (e.g. confidence intervals)
☐	☒	For null hypothesis testing, the test statistic (e.g. *F*, *t*, *r*) with confidence intervals, effect sizes, degrees of freedom and *P* value noted<br>*Give P values as exact values whenever suitable.*
☒	☐	For Bayesian analysis, information on the choice of priors and Markov chain Monte Carlo settings
☒	☐	For hierarchical and complex designs, identification of the appropriate level for tests and full reporting of outcomes
☒	☐	Estimates of effect sizes (e.g. Cohen's *d*, Pearson's *r*), indicating how they were calculated

Our web collection on statistics for biologists contains articles on many of the points above.

Software and code

Policy information about availability of computer code

Data collection	Software, version and references where necessary are described in appropriate methods section.<br>Realtime cell growth data- IncuCyte ZOOM 2018A software<br>Ligand NMR data - Topspin 2.1
Data analysis	Software, version and references where necessary are described in appropriate methods section.<br>General data and statistics- Graphpad Prism 10<br>Proteome data - MSstats v3.0,<br>Gel densitometry - Image J v1.53<br>X-ray data processing and analysis - AutoProc, Phaser, Buster, refmac, Coot and Autosolve.<br>ITC data - Origin 7.0<br>Structural alignment, RMSD calculation - Maestro, Schrödinger 2023-1 Suite<br>Multiple-sequence alignments - MAFFT<br>Realtime cell growth data - IncuCyte ZOOM 2018A software<br>Ligand NMR data - Topspin 2.1 / MestReNova 14.3.1.

For manuscripts utilizing custom algorithms or software that are central to the research but not yet described in published literature, software must be made available to editors and reviewers. We strongly encourage code deposition in a community repository (e.g. GitHub). See the Nature Portfolio guidelines for submitting code & software for further information.

Data

Policy information about availability of data

All manuscripts must include a data availability statement. This statement should provide the following information, where applicable:

- Accession codes, unique identifiers, or web links for publicly available datasets
- A description of any restrictions on data availability
- For clinical datasets or third party data, please ensure that the statement adheres to our policy

Source data are provided as a Source Data file. Atomic coordinates and structure factors of the protein-ligand complexes generated in this study have been deposited in the Protein Data Bank (PDB) under accession codes 8QM4 [http://doi.org/10.2210/pdb8QM4/pdb], 8QM5 [http://doi.org/10.2210/pdb8QM5/pdb], 8QM6 [http://doi.org/10.2210/pdb8QM6/pdb], 8QM7 [http://doi.org/10.2210/pdb8QM7/pdb], 8QM8 [http://doi.org/10.2210/pdb8QM8/pdb], 8QM9 [http://doi.org/10.2210/pdb8QM9/pdb]. The mass spectrometry proteomics data have been deposited to the ProteomeXchange Consortium via the PRIDE partner repository with the dataset identifier PXD057122 [https://proteomecentral.proteomexchange.org/cgi/GetDataset?ID=PXD057122].

Research involving human participants, their data, or biological material

Policy information about studies with human participants or human data. See also policy information about sex, gender (identity/presentation), and sexual orientation and race, ethnicity and racism.

Reporting on sex and gender	N/A
Reporting on race, ethnicity, or other socially relevant groupings	N/A
Population characteristics	N/A
Recruitment	N/A
Ethics oversight	N/A

Note that full information on the approval of the study protocol must also be provided in the manuscript.

Field-specific reporting

Please select the one below that is the best fit for your research. If you are not sure, read the appropriate sections before making your selection.

☒ Life sciences ☐ Behavioural & social sciences ☐ Ecological, evolutionary & environmental sciences

For a reference copy of the document with all sections, see nature.com/documents/nr-reporting-summary-flat.pdf

Life sciences study design

All studies must disclose on these points even when the disclosure is negative.

Sample size	Sample size generally ≥ 3, type of replicate indicated in legend, statistics only run where n>3 independent biological repeats, details of statistical test given in figure legends.
Data exclusions	No data points were excluded
Replication	Where statistical analyses were employed we selected a minimum three biological repeats to provide a basic level of replication, allowing an initial understanding of variability in the data and identifying trends while providing enough data to make statistical analyses. In addition, three repeats strikes a balance between obtaining meaningful data and managing limited laboratory time and resources. For some preliminary or exploratory experiments an n=2 was used identify trends or generate hypotheses, where necessary these were followed up with experiments with sufficient repeats for statistical analysis.
Randomization	No randomisation was used
Blinding	The plate based assays were blinded until the output of the assay were deconvoluted for data plotting.

Reporting for specific materials, systems and methods

We require information from authors about some types of materials, experimental systems and methods used in many studies. Here, indicate whether each material, system or method listed is relevant to your study. If you are not sure if a list item applies to your research, read the appropriate section before selecting a response.

Materials & experimental systems

n/a	Involved in the study
☐	☒ Antibodies
☐	☒ Eukaryotic cell lines
☒	☐ Palaeontology and archaeology
☒	☐ Animals and other organisms
☒	☐ Clinical data
☒	☐ Dual use research of concern
☒	☐ Plants

Methods

n/a	Involved in the study
☒	☐ ChIP-seq
☒	☐ Flow cytometry
☒	☐ MRI-based neuroimaging

Antibodies

Antibodies used	Monoclonal anti-eIF4E, R and D Systems Cat# MAB3228, RRID:AB_2097694.<br>monoclonal ANTI-FLAG® M2, Sigma-Aldrich Cat# F1804, RRID:AB_262044.<br>eIF4E - Cell Signaling Technology Cat# 9742, RRID:AB_823488.<br>eIF4G1 - Cell Signaling Technology Cat# 2469, RRID:AB_2096028.<br>4E-BP1 - Cell Signaling Technology Cat# 9452, RRID:AB_331692.<br>eIF4E - Cell Signaling Technology Cat# 2067, RRID:AB_2097675.<br>MCL1 - Cell Signaling Technology Cat# 39224, RRID:AB_2799149.<br>Vinculin, Cell Signaling Technology Cat# 13901, RRID:AB_2728768.<br>All used at 1:1000 except vinculin 1:2000
Validation	Antibodies were selected using https://www.antibodypedia.com/ which includes validation information. Only antibodies with peer-reviewed literature data or validated by users/supplier were selected.

Eukaryotic cell lines

Policy information about cell lines and Sex and Gender in Research

Cell line source(s)	SW620 - ATCC, cat# CCL-227, RRID:CVCL_0547<br>H1299 - ATCC, cat# CRL-5803, RRD:CVCL_0060<br>HeLA – ATCC, cat# CCL2
Authentication	All cell lines were authenticated by short tandem repeat DNA profiling
Mycoplasma contamination	All cell lines were regularly mycoplasma tested by PCR or ELISA. Only mycoplasma negative cells were allowed in our tissue culture facility
Commonly misidentified lines<br>(See ICLAC register)	N/A

Plants

Seed stocks	N/A
Novel plant genotypes	N/A
Authentication	N/A
